# Chromosomal-level reference genome assembly of muskox (*Ovibos moschatus*) from Banks Island in the Canadian Arctic, a resource for conservation genomics

**DOI:** 10.1038/s41598-024-67270-9

**Published:** 2024-09-16

**Authors:** Si Lok, Timothy N. H. Lau, Brett Trost, Amy H. Y. Tong, Tara Paton, Richard F. Wintle, Mark D. Engstrom, Anne Gunn, Stephen W. Scherer

**Affiliations:** 1 The Centre for Applied Genomics, Peter Gilgan Centre for Research and Learning, The Hospital for Sick Children, 686 Bay Street, Rm 13.9713, Suite 03-6577, Toronto, ON M5G 0A4 Canada; 2 Program in Genetics and Genome Biology, The Hospital for Sick Children, Toronto, ON M5G 0A4 Canada; 3 Program in Molecular Medicine, The Hospital for Sick Children, Toronto, ON M5G 0A4 Canada; 4 Donnelly Centre for Cellular and Biomolecular Research, University of Toronto, Toronto, ON M5S 3E1 Canada; 5 Department of Natural History, Royal Ontario Museum, Toronto, ON M5S 2C6 Canada; 6 Salt Spring Island, Canada; 7 McLaughlin Centre, University of Toronto, Toronto, ON M5G 0A4 Canada; 8 Department of Molecular Genetics, Faculty of Medicine, University of Toronto, Toronto, ON M5S 1A8 Canada

**Keywords:** *Ovibos moschatus*, Muskox, Umingmak, Continuous long read, Chromosomal-level assembly, Climate adaption, Climate change, Conservation genomics, Biodiversity, Biodiversity

## Abstract

The muskox (*Ovibos moschatus*), an integral component and iconic symbol of arctic biocultural diversity, is under threat by rapid environmental disruptions from climate change. We report a chromosomal-level haploid genome assembly of a muskox from Banks Island in the Canadian Arctic Archipelago. The assembly has a contig N50 of 44.7 Mbp, a scaffold N50 of 112.3 Mbp, a complete representation (100%) of the BUSCO v5.2.2 set of 9225 mammalian marker genes and is anchored to the 24 chromosomes of the muskox. Tabulation of heterozygous single nucleotide variants in our specimen revealed a very low level of genetic diversity, which is consistent with recent reports of the muskox having the lowest genome-wide heterozygosity among the ungulates. While muskox populations are currently showing no overt signs of inbreeding depression, environmental disruptions are expected to strain the genomic resilience of the species. One notable impact of rapid climate change in the Arctic is the spread of emerging infectious and parasitic diseases in the muskox, as exemplified by the range expansion of muskox lungworms, and the recent fatal outbreaks of *Erysipelothrix rhusiopathiae*, a pathogen normally associated with domestic swine and poultry. As a genomics resource for conservation management of the muskox against existing and emerging disease modalities, we annotated the genes of the major histocompatibility complex on chromosome 2 and performed an initial assessment of the genetic diversity of this complex. This resource is further supported by the annotation of the principal genes of the innate immunity system, genes that are rapidly evolving and under positive selection in the muskox, genes associated with environmental adaptations, and the genes associated with socioeconomic benefits for Arctic communities such as wool (qiviut) attributes. These annotations will benefit muskox management and conservation.

## Introduction

The muskox (*Ovibos moschatus*), a large iconic and charismatic animal closely related to sheep and goats within the *Caprinae* subfamily, is one of the few surviving relics of the Pleistocene megafauna^1,2^. The muskox has an intriguing recent history. It was largely unknown by the early Europeans until the first published account in 1720. For the next several decades, classification attempts were debated and confounded naturalists by the muskox’s apparent sheep, cow, and buffalo-like features^3,4^. The muskox was formally introduced into systematic zoology by Zimmermann in 1780 under the name *Bos moschatus*^5^. After further debate, it was removed from the Linnean genus *Bos* and was reassigned to a new genus, *Ovibos,* by Blainville in 1816, where the name stands today with the muskox being the only living member of that genus^6^. However, the classification debate continues to this day over subspecies designation, their number and distribution^7–9^. In the present study, we follow the last formal taxonomic revision of the species, which regarded *O. moschtus* as a monotypic species^7^.

**Figure 1:**
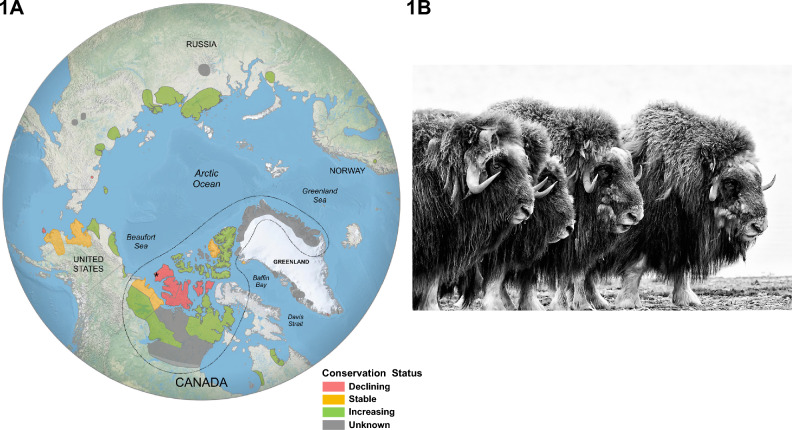
Muskox distributions. (**A**) Population distributions and regional conservation status of the muskox in 2023 (Tomaselli M, Cuyler C, Kutz S, Adamczewski J, Brodeur V, Campbell M, Cluff D, Gorn T, Hughes LJ, Leclerc L-M, Nelson M, Parr B, Sipko T, Suiter M, Taillon J and Gunn A. Muskox health, status and trends—crossing the boundaries. Unpubl. Poster, Arctic Ungulate Conference, Anchorage, Alaska, May 2023). Endemic populations in Canada and Greenland are separated from the translocated (introduced or re-introduced) populations by the enclosed dotted boundary line. The Sachs Harbour area of Banks Island, Northwest Territories, Canada, where our specimen (Royal Ontario Museum archive: ROMMRAN27; NCBI BioSample: SAMN26661894) originated is denoted by an asterisk (*). (**B**) Muskoxen in a defensive posture. Despite their well-known group defence, muskoxen are prey for wolves (*Canis lupus*) and grizzly bears (*Ursus arctos*). The muskoxen in the photograph are descendants of individuals translocated in 1935 from eastern Greenland to the Arctic Wildlife Refuge, Alaska, USA. (Photograph: ©Peter Mather, 2010).

Muskoxen are physiologically and behaviourally adapted to live year-around in the cold conditions of the Arctic tundra. Once widely distributed across the Holarctic, the present endemic population is restricted to Northern Canada and Greenland. In the last century, muskoxen have been reintroduced to Northern Quebec, Norway, Russia, Southwest Greenland, and Alaska (see Fig. 1). The reintroduction effort in Alaska was to replace populations extirpated by overhunting. Muskox populations today inhabit a large and relatively diverse range that extends from the sub-arctic northern edge of the boreal forest (56° N) to the high-arctic tundra (83° N).

Known by the Inuit as *Umingmak* (the Bearded One), the muskox is an integral and evolving part of Inuit cultural and social-economic heritage^10,11^. In addition to being a meat source, and providing direct economic benefits such as tourism and the commercialisation of hide and luxury qivut wool^12–15^, the muskox is a key species in the Arctic tundra ecosystem. Muskox grazing critically impacts plant communities and nutrient flow^16,17^ as well as carbon dioxide and methane fluxes in the Arctic biosphere^18^, which are expected to be disrupted by climate change.

The world-wide population of muskox in 2020 is estimated to be 170,000^19^. As such, the muskox holds the International Union for Conservation of Nature (IUCN) Conservation status of Least Concern^20^. However, of the 55 muskox populations reported in 2020^19^, six are in decline, including the population on Banks Island, which is well-known for its historic fluctuations in number^21^. The population on Banks Island was the largest in Canada in the 1990s but has suffered a 70% decline since 2000. Among the factors contributing to this decline include infection by a new bacterial pathogen *Erysipelothrix rhusiopathiae*, which normally infects farm animals and poultry^22,23^. Together with the expanding range of muskox lungworms^24–26^, and other diseases including brucellosis^27^, these could be early harbingers of emerging disease modalities from climate disruption.

Climate records supported by mitochondrial and microsatellite markers and by whole genome genotyping have provided evidence that the muskox underwent multiple population bottlenecks, which have resulted in contemporary populations having very low genetic diversity^9,28–30^. Although the muskox has been shown to have the lowest genome-wide heterozygosity reported in an ungulate, there are no overt signs of inbreeding depression in native muskox populations^31^. Additionally, Prewer and colleagues commented that the extremely low variability is a variance with many increases in muskox numbers and expanding distribution raising the possibility that the adaptive capacity has been under-estimated^32^. A likely mechanism is that over time, deleterious genes can be purged as have been suggested for the endangered kākāpō^33^ and the Svalbard reindeer^34^. Bayesian modelling suggests the muskox might have reached a genetic diversity minimum^32^, and it is not clear whether their remaining genetic capacity is sufficient for them to adapt to a rapidly changing Arctic.

The past decade has experienced an unprecedented increase in temperature in the Arctic. The Intergovernmental Panel on Climate Change 2023 climate models predict a mean global increase between 1.4 and 4.4** °C** by 2100^35^ with a disproportionate effect in the Arctic. Large herbivores are particularly at risk^36^. Thus, whether the muskox could physiologically and behaviourally adapt to a warmer Arctic would be of fundamental consequence for their conservation and management. An initial step to understand the muskox’s adaptive capacity has been to move toward the use of whole-genome re-sequencing^31^ based on the alignments to the draft short-read assemblies of the muskox^2,37^. While these assemblies provided an important first glimpse of the muskox genome, they are relatively incomplete and fragmented, typical of assemblies derived from short reads. As a resource for a conservation genomics-based approach for the management of the muskox, we report a chromosomal-level assembly derived from long-reads of a male muskox from Banks Island in the Canadian Arctic Archipelago. The ability of long reads to span repetitive sequences and other difficult regions of the genome^38^ has enabled the present assembly to have a more complete representation of the coding genes, isoforms of these genes, and the elucidation of their genomic organization. Moreover, the anchoring of our assembly to the 24 muskox chromosomes allows a direct cytogenetic comparison to other members of Bovidae, notably to the useful quantitative trait loci (QTLs) identified from economically important livestock. These QTLs could potentially be mapped to the muskox. From this assembly, we also provide exon-level annotation of genes that could be useful in muskox management, including genes of the immune system, those associated with environmental adaptations, particularity to heat stress, and genes associated with relevant socioeconomic benefits as direct drivers for species conservation.

## Materials and methods

### Specimen selection for genome assembly

Kidney tissue (NCBI: BioSample SAMN26661894) from a male muskox (*Ovibos moschatus*) was obtained from the Royal Ontario Museum Tissue Archive (catalog number: ROMMRAN27). The tissue was collected on 25 October 1991 near Sachs Harbor, Banks Island, NWT, Canada.

Species identity of the specimen was verified by matching a segment of the cytochrome oxidase 1 (*COX1*) gene from the specimen’s assembled mitochondrial genome against the Barcode of Life Database (http://www.boldsystems.org). The top nine hits in the database were *Ovibos moschatus* at a 100% match. Male specimens are typically selected for genome assembly to have representation of the X and Y chromosomes. The male sex of our muskox specimen was denoted by the presence in our assembly of the X and Y chromosome-specific genes, *ZFX* (Acc KAL1286804) and *ZFY* (Acc KAL1286379)^39^, and the Y chromosome-specific gene, *SRY* (Acc KAL1286397).

### DNA extraction for sequencing and genome assembly

Genomic DNA was extracted from frozen kidney tissue using a Puregene Reagent Kit (Qiagen, Hilden, Germany), with the resulting purified kidney DNA having 260 nm/280 nm and 260 nm/230 nm absorbance ratios of 1.90. DNA had a peak length of 22.6 kb on an Agilent TapeStation (Agilent, Santa Clara, CA). Although DNA from flash-frozen fresh whole blood is often preferred for long-read sequencing, when blood samples are not available due to logistical or regulatory issues, DNA purified from well-maintained archival tissue banks (particularly kidney tissues) is acceptable for PacBio sequencing and genome assembly. DNA for library construction was quantified by fluorometry using the Qubit DNA HS Assay (ThermoFisher, Waltham, MA).

### Genome sequencing and pre-assembly filtering

Long-read libraries for genome sequencing were prepared from five μg of un-sheared genomic DNA using an Express template prep kit (version 2.0) (Pacific Biosciences, Menlo Park, CA) followed by a post-library-construction sizing step of > 15 kb on the BluePippin system (Sage Science, Beverly, MA). Size-selected library was sequenced on a Sequel II sequencer (Pacific Biosciences; PacBio) in the Continuous Long-Read (CLR) mode with a 15-h movie acquisition time. Raw reads were processed on PacBio’s P filter to remove low-quality reads and adapter sequences. The longest sub-read was selected from each productive zero-mode waveguide on the flow-cell. Sub-reads of less than 2 kb length were discarded. 16,756,911 reads remained with a read length N50 value of 15,868 bp, similar to the peak length of the starting DNA (22.6 kb). Using an estimated genome size of 3 Gb inferred by tabulating k-mer frequencies^40^, the resulting CLRs used for the assembly comprised 207.6 Gb of sequences, representing 71.6× genome coverage. Read statistics, including genomic coverage at different ranges of read-length N50 values are provided in Supplementary Figure S1.

Short reads used to polish the assembled genome were produced from 700 ng of the same genomic DNA used for long-read sequencing. DNA was randomly fragmented to 500–600 bp using a Covaris LE220 Focused Ultrasonicator (Covaris, Woburn, MA), followed by library construction carried out in accordance with the Illumina TruSeq DNA PCR-Free protocol (Document 1000000039279v00) (Illumina, San Diego, CA). Sequencing was performed on the HiSeq X sequencer (Illumina), producing 2 × 150 nt paired-end reads. Only sequences with quality scores Q35 or better were used. To achieve this level of accuracy, the first 10 bases were discarded in read 1 and read 2, the last 20 bases were discarded in read 1, and the last 30 bases were discarded in read 2. After trimming the least accurate portions of the reads and removal of adapter sequences, 777 million paired-end reads remained, comprising 178.6 Gb of sequences (61.6× coverage).

### De novo genome assembly and chromosome assignment

A two-step assembly workflow was used to assemble the muskox genome. In the first step, de novo genome assembly was carried out directly from uncorrected PacBio CLRs (71.6× coverage) using Flye 2.8.2, which was designed to assemble error-prone reads^41^. To resolve mis-assemblies and other errors, the primary assembly was polished three times with Flye-polish^41^ using the same CLRs that were used to construct the assembly. To correct residual PacBio sequencing errors remaining in the primary assembly, typically single base or small insertions and deletions (indels) inherent to CLRs, the assembly was subjected to eight rounds of additional polishing with high-quality trimmed Illumina short-reads (61.6× coverage) using Freebayes 1.3.1 (https://github.com/freebayes/freebayes).

In the second step, multiple cross-species scaffolding against reference genomes of related Cervidae was used to generate super-scaffolds to produce a chromosomal-level assembly for the muskox. To mitigate potential bias during this process, all input de novo assembled contigs and scaffolds of the muskox were to be left intact and unaltered. This cross-species super-scaffolding step drew heavily on the previous comparative studies of karyotypes in the Cervidae family^42^ and on the comparative chromosome painting-based map of the muskox, dromedary (*Camelus dromedaries*) and human^43^, and similar information from 43 other bovid genomes^44^. The chromosome number of the muskox was established at 2n = 48^43,45,46^. Making use of the finding that autosomal arms are highly syntenic in typical Bovidae family members^42,47^, we used the chromosomal-level reference genomes of the closely related takin (*Budorcas taxicolor*) (GCF_023091745.1), goat (*Capra hircus*) (GCF_001704415.2), and sheep (*Ovis aries*)(GCA_016772045.2) to scaffold our muskox contigs into provisional chromosome arms. Muskox contigs were aligned against the aforementioned reference genomes using minimap2 (v2.24)^48^ with parameter − x asm20, allowing alignments with up to 20% divergence in the nonrepetitive sequences^49^. As a quality control step, the resulting alignments were then reviewed and filtered to ensure there were no contradictions with the Repeat Graph generated by the Flye assembler. The Repeat Graph represented all assembled contigs and their junctions, thus providing a guide as to whether viable paths between contigs in question were plausible during scaffolding. The resulting scaffolded chromosome arms were then localised and assigned to 24 provisional chromosomes using the cattle genome (GCF_002263795.3) as an anchor in accordance with the cross-species relationships previously established^43^. Chromosomal scaffolding, orientation and assignments were further refined and adjusted with a final alignment against the comparative chromosome map established by chromosome painting for the muskox, human (GCF_000001405.40) and the dromedary genomes (GCF000803125.2)^43^. In another quality control step, Minimap2 was used once again to return the best pairwise unique matching segments against the human and dromedary genomes, enabling our assembled scaffolds to be anchored to the 24 chromosomes of the muskox with high confidence. In a final quality control step, long reads were tiled along the length of the assembly to identify regions of discordance for curation. The resulting assembly, O.moschatus_RAN27-v1.0, has a contig N50 of 44.7 Mbp, a scaffold N50 of 112.3 Mbp, and a completeness of 93.49% as determined by the tabulation of k-mers derived from highly accurate Illumina short-reads using Merqury^50^.

### Sequence contamination in muskox assembly

As PacBio CLRs could have up to 15–20% pseudorandom errors in the forms of base substitutions and small insertions and deletions^51^, a search for DNA contamination from environmental or laboratory sources might not be sensitive at the read level. Likewise, Illumina short reads might be too short to make reliable and unambiguous calls of potential contaminating DNA. As a consequence, contamination assessment was carried out in the final assembly after the ensuring correction steps for sequencing errors.

Potential microbial and other environmental DNA contaminants in the DNA sample that made it into the final assembly were assessed using the workflow depicted in Supplementary Fig. 2. BLAST analysis of non-overlapping 5 kb windows in the assembly revealed five windows showing a match (95–99%) to *Delftia acidovoran* (Acc CP058970.1), a common aerobic environmental organism found in soil. The five positive windows were adjacent to each other and were all confined to a single contig of 26 kb length, which was removed from the assembly. No microbial or fungal sequences were found amongst the top 50 ranked hits in the remaining windows, indicating there were no other overt environmental contaminants in the final muskox assembly.

Human DNA contamination has been reported in many non-primate genome databases, presumably from laboratory sources^52^. Using a primate-specific SINE, *AluY*, we found no discernible primate sequences in the muskox assembly.

### BUSCO and genome annotation

The completeness our muskox assembly was qualitatively assessed using BUSCO v5.2.2 (Benchmarking Universal Single Copy Orthologs; mammalia_odb10)^53^. BUSCO designates the status of a set of 9226 mammalian lineage marker genes as complete, fragmented, or missing in an assembly. Muskox genes that were failed by BUSCO were subjected to a verification step at exon-level resolution to determine their true status in our assembly. In this verification procedure, we first identified a seed exon for that the gene query in the assembly using a set of orthologous human, sheep, and cow exon probes (NCBI RefSeq). Typically, the seed exon has the highest BLAST score amongst the exon queries for the gene under investigation. From the seed exon, we then scanned up-stream and down-stream along the assembly or scaffold using BLAST in incremental 10 kb size windows in the search for the adjacent exon. Specific search criteria include nucleotide and amino acid sequence identity to the query exon, and the conservation of the reading frame relative to the consensus splice signals at the exon boundaries. Once an adjacent exon was identified, the process was repeated until all exons were identified. For a gene to be designated as complete in the muskox assembly, the full-length polypeptide encoded by the predicted exons must begin with an initiation codon and end with a termination codon, and to be at least 70% identical to the full-length human, sheep, takin, or cow protein encoded by the query as reported in RefSeq.

### Correction of sequencing errors

Sequencing errors in the PacBio platform are typically between 10 and 15%, comprising single base substitutions or small indels. These errors were corrected in the primary assembly using Flye-polish^41^ followed by eight rounds of “polishing” with high-quality trimmed Illumina short-reads of Q35 or better using Freebayes 1.3.1 (https://github.com/freebayes/freebayes). From monitoring the kinetics of base changes in the primary assembly at different rounds of polishing, we found base changes from polishing with Freebayes do not approach a plateau value until after the fifth or sixth rounds, after which further base changes are confined to a small number of positions where they alternate between what appear to be allelic bases in successive rounds (data not shown).

### Assessment of residual sequencing errors in the assembly

In the absence of a reference DNA of a known sequence to make a direct comparison, it was not possible to tabulate the base accuracy of our assembly following the polishing steps. However, we can tabulate base substitute errors indirectly when errors generate premature termination codons disrupting the Open Reading Frames (ORFs) we have annotated.

The following formula depicts the probability of creating a spurious termination codon at different levels of sequencing accuracies:

$$T = \left( {1 - A^{3} } \right) \times 3/63$$

where *A* is the probability of a correct base call (i.e. the sequencing accuracy for that nucleotide position); *A*^3^ is the joint probability of three consecutive correct calls forming a correct codon; 1 − *A*^3^ is the probability of a codon being incorrect with at least one incorrect base call; 3/63 is the probability of an incorrect codon being a termination codon*; *T* is the probability of an in-frame termination codon created from an in-frame non-termination codon due to sequencing error; * Of the 64 possible codons, only one is correct and 63 are incorrect at a given position. Since there are three possible termination codons, therefore, the probability of an incorrect codon being a termination codon is 3/63.

The muskox gene with the longest coding capacity, *Titin* (*TTN*, Acc KAL1287845), in conjunction with the annotated genes in Supplementary Tables S2, S3, S8, and S9, yield ORFs comprising 782,521 codons. Since all of these genes were found to encode uninterrupted ORFs where none of the 782,521 codons are termination codons, a value for *T* can be set at < 1/782,521 or < 0.00013%. Substituting this value for *T* and solving for *A* in the aforementioned formula yields an inferred sequence accuracy in the assembly of not less than 99.99910%, corresponding to a Phred score^54^ of no less than 50.4839 in these coding regions.

### Identification of repetitive DNA in the genome

Repetitive elements described in RepBase were identified and tabulated in the muskox genome using RepeatMasker^49^. Supplementary Table S1 summarizes the distributions of repetitive sequences in the muskox.

### Assessment of genetic diversity in muskox

Genetic diversity was assessed for the Banks Island muskox specimen assembled in this study, and was compared to the genetic diversity of selected muskox specimens collected by Van Coeverden de Groot and colleagues^55^ and Pečnerová and colleagues^31^. Ilumina short reads from selected specimens with the highest sequence read depth were downloaded from NCBI SRA (see Supplementary Table S11 for accession numbers) from each geographic region depicted in Fig. 1 of Pečnerová’s paper^31^. Reads from the selected specimens comprised an average 14× coverage of the muskox genome (median coverage of 12×).

Sequence reads were aligned to the muskox reference genome in the present study using BWA 0.7.17r1198^56^. Duplicate reads were marked using the “MarkDuplicate” function of GATK 4.1.9.0^57^. The average depth of coverage of each sample was calculated using the “depth” function of SAMtools 1.9^56^. To ensure that heterozygosity estimates were not confounded by differences in read depths, reads from samples with high read depths were subsampled to 10–15× average depth. Single nucleotide variants (SNVs) and small insertions and deletions (indels) were detected using Genome Analysis Toolkit (GATK) best practices^58^.

Using the alignment file from BWA as input, SNVs and indels were identified using the “HaplotypeCaller” function of GATK with parameter—minimum-mapping quality 20 followed by the GATK function “GenotypeGVCFs”. The FILTER column of the VCFs was populated using the hard-filtering criteria suggested by the authors of GATK (https://gatk.broadinstitute.org/hc/en-us/articles/360035890471-Hard-filtering-germline-short-variants). For SNVs the criteria were: QD < 2.0, MQ < 40.0, SQR > 3.0, SOR > 3.0, FS > 60.0, MQRankSum < − 12.5, and ReadPosRankSum < − 8.0. For indels the criteria were: QD < 2.0, SQR > 10.0, FS > 200.0, and ReadPosRankSum < − 20.0. Only variants passing these filters were used for assessing heterozygosity or to make variant calls against the reference muskox genome. The degree of heterozygosity for each genome was tabulated based on the number of heterozygous variants per megabase of reference sequence. To assess genetic diversity in the muskox immune genes including genes in the MHC, we determined the exon coordinates for the genes of interest and tabulated the calls within those coordinates from the alignment files for each muskox specimen, as well as the variant counts and profiles aggregated across all of the specimens.

### Mitochondrial genome assembly

The muskox mitochondrial genome was assembled with Abyss V2.1.5 (parameters k = 103, kc = 6)^59^ from 40 million Illumina paired-end reads. The NCBI muskox mitochondrion sequence NC_020631.1 was used as a BLAST query to select the contig containing the presumptive mitochondrion sequence. Circularization of the selected contig yielded a complete mitochondrial genome of 16,431 bp. The accuracy of the mitochondrial assembly was supported by the uninterrupted tiling of mapped Illumina short reads across the length of the assembly in 50 bp moving windows of single base increments. Tiling depths of greater than 7500 reads were tabulated across 100% of the assembled circular mitochondrion genome. The mitochondrial assembly was further supported by the alignment of PacBio long reads, yielding a read depth of 90×. We also performed a de novo assembly of the aligned long reads and produced a circular contig of 16,341 bp in length that is identical to the one assembled from short reads.

## Results and discussion

### Assembly strategy and assessment of assembly quality

Pacific Biosciences (PacBio) Continuous Long-Reads (CLRs) were assembled directly using the Flye assembler^41^ into a high-quality haploid assembly for the muskox. The resulting assembly, O.moschatus_RAN27-v1.0, has a contig N50 of 44.7 Mbp, a scaffold N50 of 112.3 Mbp, and a completeness of 93.49% as determined by the tabulation of k-mers derived from highly accurate Illumina short-reads using Merqury^50^. The incomplete portion of the assembly can be attributed to the incomplete Chromosome Y, centromeres, telomeres, and other unresolved repeats that are missing in the assembly.

As shown here and in our previous study of the North American wolverine genome^60^, the 15–20% pseudorandom sequencing errors typically associated with CLRs^51^ can be effectively mitigated by the Flye assembler and the ensuing polishing regimens to yield a high-quality assembly that is essentially free of residual sequencing errors. Using the assessment method for the estimation of residual sequencing errors described in Materials and Methods, we estimate the sequence accuracy in our muskox assembly after the polishing steps to be not less than 99.99910% corresponding to a Phred score of not less than 50.4839 (corresponding to < 1 substitution error in 111,787 bases in the coding regions). This level of accuracy exceeds the recommend value for “finished genomes”^61^.

We favour the error mitigation strategy used in this study over the computationally intensive approach of correcting individual PacBio reads prior to assembly^62^ or the use of PacBio’s recent Circular Consensus Sequencing also known as HiFi Sequencing (HiFi/CCS)^63,64^ where reads are partially corrected during sequencing. HiFi/CCS holds promise as the technology continues to improve in accuracy and achieves a more favourable cost profile; however, at the time of this study, HiFi/CCS technology could achieve a uniform Q30 or better correction for only a small portion of the flow cell, with the majority of the other reads having very heterogeneous and lower degrees of correction. This heterogeneity in read accuracy complicates the models used by many assemblers to build contigs. Moreover, the HiFi/CCS process itself often has a detrimental effect on the overall lengths of the reads compared to CLR, with concomitant impact on the efficiency of scaffolding and assembly. By contrast, assemblers such as Flye could easily model the uniform, but albeit higher overall error rate across all CLRs, to build accurate contigs. At least at the time of this study, CLRs arguably offer a better functional trade-off for cost effective de novo assembly of large, complex genomes.

Table 1 compares the assembly metrics of our muskox assembly, O.moschatus_RAN27-v1.0, against the two current short-read assemblies for the muskox (ASM2148233v1)^2^ and (ASM2253363v2)^37^. All three muskox assemblies have similar ungapped lengths of between 2.5 and 2.6 Gb. The improved continuity of our long-read assembly is evident by having a much higher contig and scaffold N50 values and lower contig and scaffold counts over the previously reported short-read assemblies for the muskox and other ruminant genomes assembled from short reads^65^. Of the two short-read assemblies for the muskox, the assembly reported by Prewer and colleagues^37^ is more fragmented, which could be due to the lower quality of the source DNA extracted from hide as opposed to the DNA extracted from fresh blood used by Li and colleagues^2^. The low contig and scaffold L50 values (i.e., the number of contigs or scaffolds that represent half the genome) further reflect the improved contiguity offered by long-read technologies. In terms of the usual continuity metrics, our muskox assembly is at par with, or is better than, the other chromosomal-level reference assemblies currently available for Bovidae, the takin, goat, sheep, and cow.

**Table 1:** Assembly metrics.

	*Ovibos moschatus* (Muskox) male 2024/04/26 O.moschatus _RAN27_v1.0	O. moschatus* (Muskox) male 2021/01/13 ASM2146233v1	*O. moschatus* (Muskox) male 2022/05/16 ASM2253363v2	Budorcas taxicolor* (Takin) male 2022/04/22 Takin1.1 ARS-UI_Ramb_v3.0	Ovis aries* (Sheep Breed: Rambouillet) female 2023/07/20 ARS1.2	Capra hircus* (Goat) male 2016/08/24 ARS-UCD2.0	Bos taurus* (Cattle Breed: Hereford) female 2023/07/01
Total sequence length (bp)	2,602,202,933	2,615,835,778	2,621,888,837	2,851,954,952	2,654,063,983	2,922,617,086	277,0686,120
Total ungapped length (bp)	2,602,159,733	2,601,841,192	2,473,402,365	2,851,579,369	2,654,021,983	2,922,578,899	2,770,657,958
Number of contigs	654	14,255	114,188	1515	227	30,378	2344
Contig N50 (bp)	44,730,146	843,055	38,369	68,053,581	43,178,051	26,244,591	26,402,946
Contig L50	20	832	18,652	15	24	32	32
Number of scaffolds	222	6709	8658	1363	143	29,886	1958
Scaffold N50 (bp)	112,334,376	46,819,416	13,200,690	109,747,698	101,274,418	87,277,232	103,308,737
Scaffold L50	9	20	61	10	8	13	12
	72× PacBio Sequell II CLR Flye 2.8.2 Freebayes 1.3.1	60× Ilumina Hi-Seq SOAPdenovo 1.12 (Li et al.^2^)	85× Ilumina Hi-Seq SOAPdenovo 240 (Prewer et al.^37^)	24× PacBio Sequel I CLR Illumina NovaSeq Bionano Hifiasm v0.13	55× PacBio RSII Nanopore Illumina HiSeq Canu 1.8 Nanopolish 0.12.5 Salsa 2.2 PB Jelly 15.8.24 Freebayes 1.3.1	50× PacBio Bionano Hi-C Celera 8.2 Bionano Irys April 2015 Lachesis HiC June 2015	Various Falcon v FEB-2016

Comparison of the muskox assembly of this study with other muskox and Bovidae assemblies. Asterisk (*) denotes current NCBI reference genomes.

### Assembly completeness and residual sequence errors

**Figure 2:**
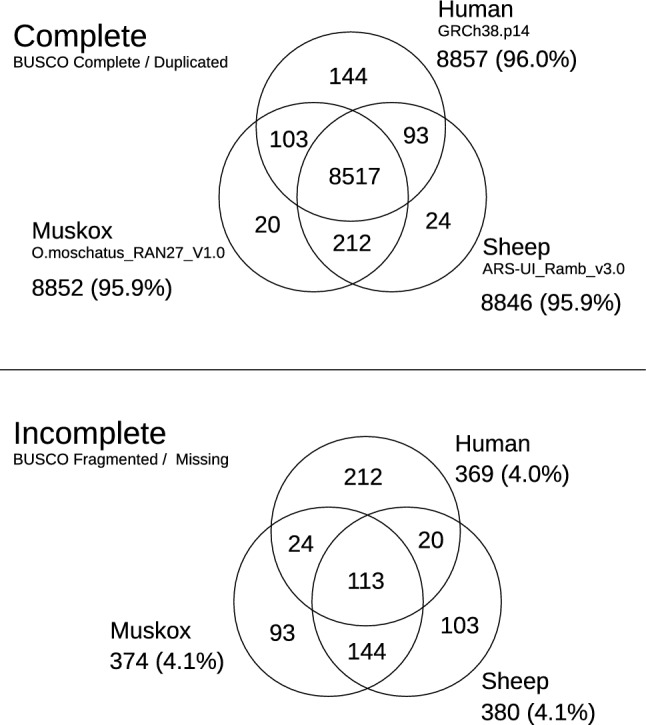
Venn diagram of BUSCO results. Analysis by Benchmarking Universal Single Copy (BUSCO) v5.2.2 of the muskox O.moschatus_RAN27_v1.0, human GRCh38.p14 (GCF_000001405.40) and sheep ARS-UI_RAMb_v3.0 (GCF_016772045.2) assemblies. The mammalian linage (mammalia_odb10) comprised of 9226 ortholog groups was used. “Complete genes” refers to those scored as “Complete” or “Duplicated” by BUSCO, while incomplete genes refer to those scored as “Fragmented” or “Missing” by BUSCO. The official human reference GRCh38.p14 only had 96.0% BUSCO complete even though it is the most thoroughly annotated genome to date. All muskox incomplete genes were found and annotated in the muskox assembly (Supplementary Table S2).

The BUSCO (Benchmarking Universal Single-Copy Orthologs) program has long been used to assess the completeness of genome assemblies^53^. When BUSCO v5.2.2 with its set of 9226 mammalian linage marker genes was applied to our muskox assembly, 8852 genes (95.9%) were scored as complete, and 374 genes (4.1%) were scored as fragmented or missing in the assembly. At face value, these BUSCO scores placed our muskox assembly in the top echelon for reported genome assemblies derived from long reads. Figure 2 compares the BUSCO scores of our muskox assembly with current NCBI designated reference genome assemblies for sheep (ARS-UI_Ramb_v3.0; GCF_016772045.2) and human (GRCh39.14; GCF_000001405.40).

An important and useful feature of BUSCO is its ability to assess the completeness of genome assemblies across a broad range of species. This was accomplished by balancing sensitivity and specificity to create a single universal set of consensus gene profiles that can be used generically across all mammalian species. To enable this feature, gene profiles are not optimized for any particular species. Due to this compromise, it is possible that subsets of BUSCO profiles could have difficulties detecting their cognate genes in species whose orthologs deviate significantly from the consensus. Accordingly, BUSCO could under-count those genes, resulting in an under-estimation of the quality of an assembly being evaluated. Undercounting of genes by BUSCO was exemplified by our recent genome assembly for the wolverine^60^ and by the current build of the gold standard human reference genome, GRCh38.p14, where 369 BUSCO genes were called as fragmented or missing (Fig. 2), despite those genes being annotated by the NCBI Genome Data Viewer as complete and experimentally verified.

To address the possibility of an undercounting in our muskox assembly, the 374 genes that BUSCO scored as fragmented or missing (Fig. 2) were annotated at exon-level resolution. The results revealed that all 374 genes are present and complete (Supplementary Table S2), revising the BUSCO score to 100%. Interestingly, amongst the genes BUSCO deemed to be missing in the muskox, *LRP1B* (LDL receptor related protein 1B), might have evaded BUSCO solely due to its size. In many species, *LRP1B* is among a small number of genes that span more than a million base pairs (2.20 Mbp in sheep and 1.90 Mbp in human). In the muskox, *LRP1B* (Acc KAL1287620) comprises 91 highly conserved exons spanning 2.17 Mbp, encoding 4596 amino acids, and is present in its entirety on contig MUS01_AAA20220901_F8-ctg00025 (Acc JBFTXH010000025).

### Assessment of genome-wide genetic diversity

**Figure 3:**
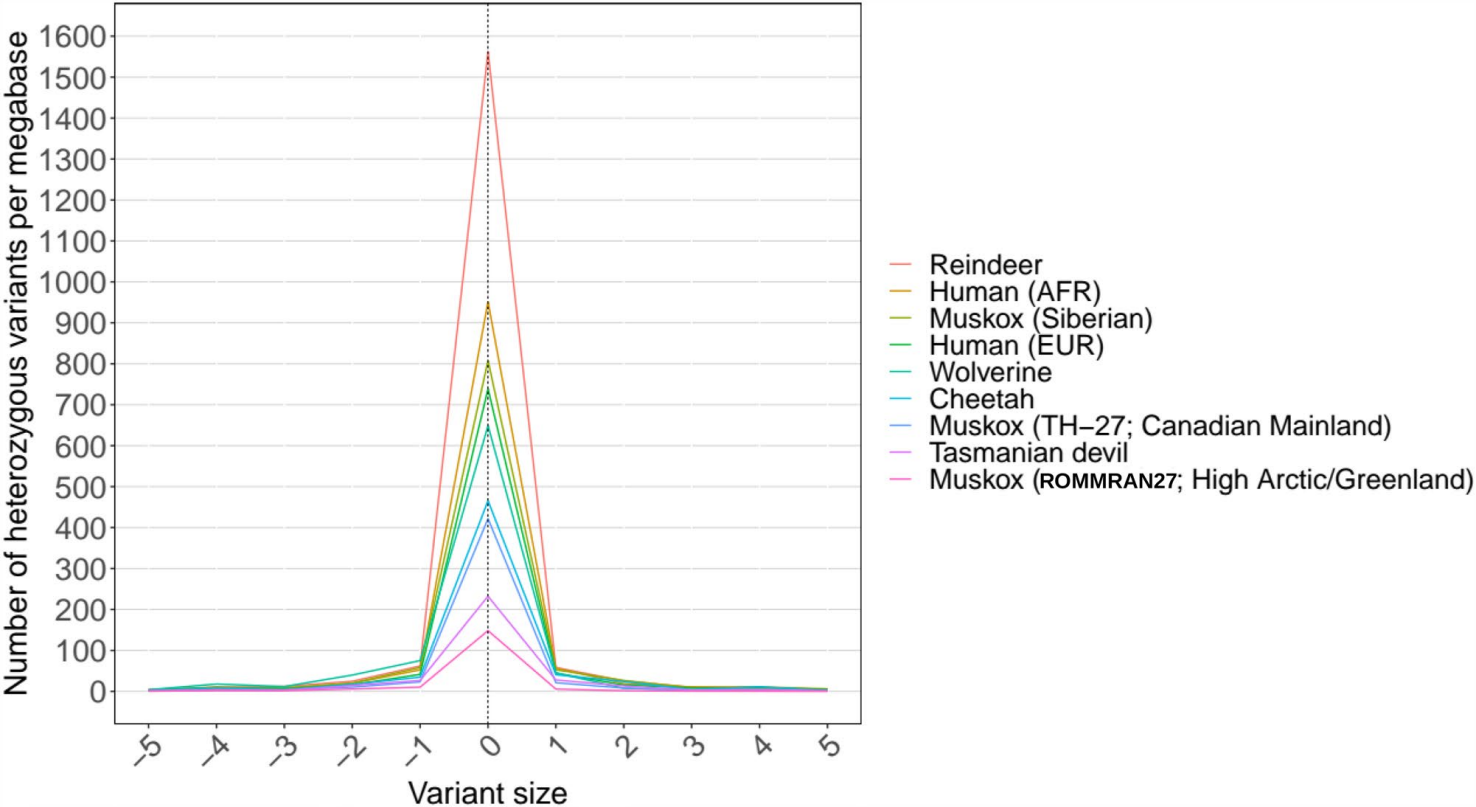
Number of heterozygous variants per megabase of reference sequence for variants of various sizes. Negative values on the x-axis indicate deletions, positive values indicate insertions, and a value of zero indicates single nucleotide variants. Heterozygous variant frequencies of different muskox populations are plotted against two diverse outbred species (human of African and European descent and wolverine), and two species that had undergone recent genetic bottleneck, the cheetah and the Tasmanian devil. The heterozygosity of the Canadian mainland muskox is on par with the cheetah, while that of the High Arctic/Greenland muskox is lower than that of the Tasmanian devil. The other large Arctic ruminant, the reindeer, is relatively diverse in comparison.

Genetic heterozygosity was tabulated for our specimen and compared to the muskox previously sampled across Greenland and Canada^31,55^. Figure 3 depicts plots of the number of heterozygous variants per megabase for the muskox, two threatened mammalian species, and a ~ 21,000-year-old ancient muskox sample from Siberia. Consistent with previous findings measured by the tabulation of heterozygous positions, contemporary muskoxen have very low overall genetic diversity across the genome, although mainland muskoxen have higher diversity than those from the Arctic Islands, possibly because the muskox survived the 1900s over-harvesting in six geographically separated refugia. The ancient sample from Siberia showed that genetic diversity was higher in the past. Muskox genetic diversity is lower than two mammalian species under threat that have undergo recent genetic bottlenecks, the Tasmanian devil (*Sarcophilus harrisii*)^66^ and the cheetah (*Acinonyx jubatus*)^67^. Interestingly, despite the muskox having the lowest genome-wide heterozygosity reported in an ungulate, there are no overt signs of inbreeding depression in native muskox populations^31^. The other large Arctic ruminant, the reindeer, is relatively diverse in comparison.

### Muskox subspecies

Although muskoxen have not been viewed to be sufficiently variable to warrant sub-species recognition^7^, recent genome analysis showed a distinct clustering of barren-ground muskoxen separate from the Greenland and Arctic Island muskoxen^9,31^. However, the interpretation of the taxonomic significance of this clustering pattern is not clear in a species with such low genetic diversity. Moreover, phenotypic differences due to diet and nutrition, age, or other environmental factors have not been rigorously investigated. As such, the designation of muskox subspecies is still an open question awaiting more studies.

### Muskox mitochondrial genome assembly and nuclear mitochondrial DNA

Identical, full-length mitochondrial genome sequences of 16,431 bp were assembled from long and short reads (Acc CM083088.1). Sequence comparisons with the other muskox mitochondrial genomes reported showed only minor differences, notably in the control-region, consistent with the muskox being monotypic, and do not allow definition of muskox subspecies^30^.

Blast analysis of the muskox mitochondrial genome against the O.moschatus_RAN27_V1.0 assembly at a threshold of 1E4 revealed 597 potential mitochondrial pseudogene loci of varying lengths and degeneracies (data not shown). These “Nuclear Mitochondrial DNAs” (NUMTs), are believed to originate from invasion of the nuclear genome by mitochondrial DNA via nonhomologous recombination^68^. It is generally viewed that the accumulation of NUMTs is a continuous evolutionary process^69^. Using the same threshold, the mouse genome (*Mus musculus*) has 190 copies, the rat genome (*Rattus norvegicus*) has 61 copies, and the human genome has 1356 copies^68^.

### Chromosome assignment and chromosome evolution

**Figure 4:**
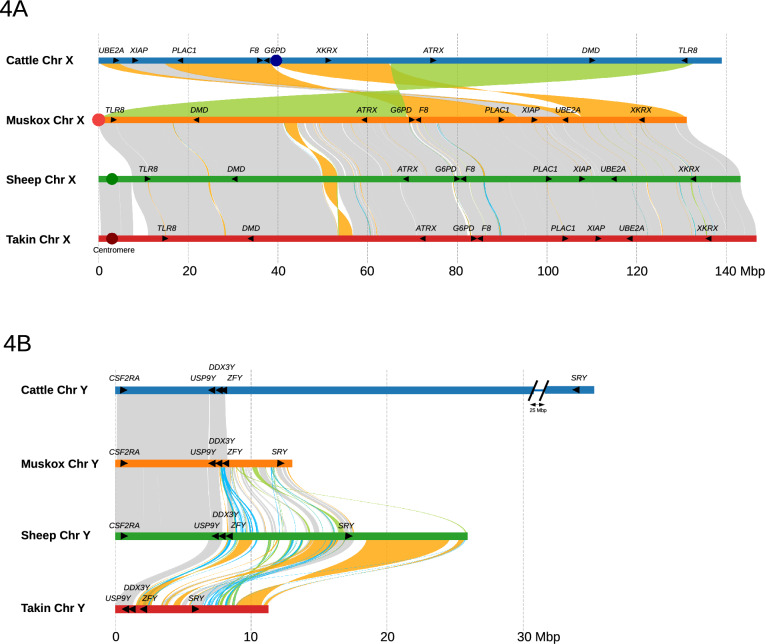
Synteny of the sex chromosomes between muskox, cattle, sheep and takin. Grey-shaded ribbons indicate regions of broad synteny, orange ribbons represent inversions, green ribbons represent translocations, and blue ribbons indicates duplications. Plotsr^71^ was used in the construction of the figure. (**A**) Comparison of chr X between the four animals. The cattle differs from the rest by three inversions and one translocation with inversion. The sheep mainly differs from muskox and takin by one inversion. The beginning of sheep and takin chromosome X represents a centromeric region not assembled in muskox. Selected genes associated with X-linked disorders or discussed in the paper are marked along with their orientation. (**B**) Comparison of chromosome Y between the four animals. Chromosome Y proved challenging to assemble and remained highly fragmented and incomplete for muskox, sheep and takin. Selected Y-specific genes were annotated and marked denoting their orientation.

As described in the Materials and Methods, we drew heavily on the previous comparative studies of karyotypes in the Cervidae family^42,43^ to anchor our assembly to the 24 chromosomes of the muskox. The muskox karyotype differed from the ancestral pecoran karyotype by six major fusions, one fission, and three inversions. The muskox karyotype includes six submetacentric and 17 acrocentric autosomes, and the sex chromosomes. Our assembly provides near complete representation of all the autosomes. The assembly of the X chromosome is essentially complete. However, the assembly of the Y chromosome is fragmented due to the extremely high composition of repetitive DNA, despite the use of the current generation of long reads. Further resolution of the Y chromosome would require yet longer and more accurate reads, perhaps combined with optical mapping. The comparative assemblies of the X and Y chromosomes in the muskox, takin, sheep and human are shown in Fig. 4. Table 2 presents the correspondence between conserved chromosome segments in the muskox, takin, sheep, goat, cow, pecoran ancestral karyotype^70^, and human.

**Table 2:** Correspondence of conserved chromosomal segments between muskox, sheep, goat, takin, pecoran ancestral karyotype (PAK), cattle and human.

MUSKOX	Sheep (GCA_016772045.2)	Goat (GCF_001704415.2)	Takin (GCF_023091745.1)	Pecoran Ancestral karyotype (PAK)	Cattle (GCF_002263795.3)	Human (GCF_000001405.40)
1p	17	17	17	N1	17	22q′/12q″/4pq
1q	1q	1	1q	A2	1	21/3/21
2p	20	23	11p	R	23	6p
2q	2q	2	2q	B2	2	2q″/1
3p	24	25	2p	T	25	16p/7
3q	3q	5	5q	C2	5	12pq′/22q″/12pq′/22q″
4p	26	27	24	V	27	4/8p″
4q	3p	11	11q	C1	11	2pq/9
5p	21	29	25	W	29	11
5q	23	24	22	s	24	18
6p	25	28	5p	u	28	10q
6q	19	22	1p	Q	22	3
7	6	6	6	F	6	4pq
8	5	7	7	E	7	19p/5
9	1p	3	3	A1	3	1
10	2p	8	8	B1	8	8p7 9/8p′/9
11	12	16	16	K	16	1
12	4	4	4	D	4	7
13	7	10	10	G	10	15/14/15/14
14	8	9	9	H1	9	6q
15	14	18	18	M	18	16q/19q
16	18	21	21	P	21	15/14
17	10	12	12	I	12	13
18	13	13	13	J	13	20/10p/20
19	15	15	15	L	15	11
20	11	19	19	N2	19	17
21	9	14	14	H2	14	8q
22	16	20	20	0	20	5
23	22	26	23	U	26	10q
X	X	X	X	X	X	X
Y	Y		Y		Y	Y

The relationships between muskox, PAK, cattle, and human were established by Proskuryakova and colleagues through chromosome painting^43^ and validated in this study by sequence alignment. The correspondence between muskox and sheep, goat and takin were established in this study by sequence alignment. Corresponding segments were ordered beginning from the centromere (most relevant for the human column). Chromosome 7–23 and X in muskox are acrocentric. The karyotype of muskox represents a departure from the PAK by six fusions (N1 + A2, R + B2, T + C2, V + C1, W + S, U + Q) and one fission (U), maintaining an autosomal fundamental number (i.e. autosomal arm count) of 58, a stable number for most karyotyped Bovidae species with varying diploid chromosome number (2n).^47^.

### Conservation genomics

Global ecosystems are under threat from rapid climate change^35,72^. In addition to direct threats to food supplies, and alterations to prey-to-predator or host-to-pathogen dynamics, there are climate induced anthropogenic disruptions affecting the Arctic ecology^73^. The latter include increased human intrusions and industrial activities. Large herbivores, such as the muskox, are particularly at risk from such ecological disruptions^19,36^.

Arctic animals are adapted to live year-round in extreme seasonal variations^2,34,74–76^, characterized by a short season of plant growth and a period of less digestible forage^19^. Temperature (and duration) and photoperiod changes are major cues regulating host–pathogen interactions^77^. The disruption of these seasonal cues by climate change could have profound consequences in host–pathogen dynamics in the Arctic^78–81^.

Conservation genomics is application of advanced genomics analyses for the preservation of the viability of populations and the biodiversity of living organisms. This important arsenal of tools helps to mitigate the effects of climate change^82–87^. High-quality reference genome assemblies are increasingly recognized as an important foundation of conservation genomics^84–91^. This is particularly so as the new chromosomal-level assemblies derived from long reads are superseding the earlier, more fragmented assemblies with fewer gene representations.

One immediate use for the new assemblies is for the identification of comprehensive sets of relevant genes at exon level resolution. These gene sets are the starting points to identify specific genes or variants to elucidate adaptive pathways, and to develop genomic and epigenomic biomarkers to monitor gene flow, hybridization and introgression, disease susceptibility, and other responses to a changing environment. To provide this important resource for the muskox, we have annotated selected families or classes of genes that could participate in the conservation and management of Arctic wildlife. These include genes encoding components of the innate immune system, and are provided in Table 3 and in Supplementary Tables S3 through S9.

**Table 3:** Selected genes of the innate immunity pathways.

Gene symbol	Alternative name/symbol	Accession	Muskox chromosome
**Toll-like receptors**			
*TLR1*	*CD281*	KAL1288282	Chr 7
*TLR2*	*CD282*	KAL1286936	Chr 1
*TLR3*	*CD283*	KAL1287412	Chr 4
*TLR4*	*CD294*	KAL1288035	Chr 10
*TLR5*	*CD295*	KAL1288066	Chr 11
*TLR6*	*CD296*	KAL1288284	Chr 7
*TLR7*	*CD287*	KAL1286564	Chr X
*TLR8*	*CD288*	KAL1286565	Chr X
*TLR9*	*CD289*	KAL1287817	Chr 6
*TLR10*	*CD290*	KAL1288283	Chr 7
**Nod-like receptors**	**NLR family pyrin domain containing**		
*NLRP1*	*NALP1; CARD7*	KAL1287737	Chr 20
*NLRP2*	*NALP2*	KAL1286518	Chr 15
*NLRP3*	*NALP3*	KAL1288227	Chr 8
*NLRP5*	*NALP5*	KAL1286519	Chr 15
*NLRP6*	*NALP6*	KAL1286925	Chr 5
*NLRP8*	*NOD16*	KAL1286520	Chr 15
*NLRP12*	*NALP12*	KAL1286516	Chr 20
*NLRP13*	*NOD14; NALP13*	KAL1286517	Chr 15
*NLRP14*	*NALP14*	KAL1287893	Chr 19
*NLRX1*	*NOD5*	KAL1287894	Chr 19
*NOD1*	*NLRC1; CARD4*	KAL1288359	Chr 12
*NOD2*	*NLRC2; CARD15*	KAL1287942	Chr 15
*NLRC3*	*NOD3*	KAL1287486	Chr 3
*NLRC5*	*NOD4*	KAL1287941	Chr 15
**C-type receptors**	**C-type lectin domain family**		
*CD209*	*CLEC4L; DC-SIGN*	KAL1286854	Chr 8
*OLR1*	*CLEC8A*	KAL1288126	Chr 3
*CLEC1A*	*CLEC1*	KAL1288098	Chr 3
*CLEC1B*	*CLEC2; CLEC2B*	KAL1288099	Chr 3
*CLEC4D*	*CD368; CLEC6; CLECSF8; DECTIN-3*	KAL1288101	Chr 3
*CLEC4E*	*CLECSF9; MINCLE*	KAL1288102	Chr 3
*CLEC4G*	*DTTR431*	KAL1286857	Chr 8
*CLEC5A*	*CLECSF5*	KAL1287060	Chr 12
*CLEC7A*	*CLECSF12; DECTIN-1*	KAL1288104	Chr 3
*CLEC9A*	*CD370; DNGR-1*	KAL1288105	Chr 3
*CLEC12A*	*CD371*	KAL1288097	Chr 3
**Rig-like receptors**	**Retinoic acid-inducible gene-l-like receptors**		
*RIGI*	*DDX58; DExD/H-box helicase 58; RLR1*	KAL1287283	Chr 10
*DHX58*	*RLR-3; LPG2; RLR3*	KAL1286474	Chr 20
*MAVS*	*CARDIF; IPS-1; IPS1; VISA*	KAL1287541	Chr 18
*I* *FIH1*	*MDA5; IDDM19; RLR; 2IDDM19*	KAL1287835	Chr 2
*ZBP1*	*Z-DNA binding protein 1*	KAL1287555	Chr 18
**Adaptors**			
*MYD88*	*IMD68; MYD88D*	KAL1287811	Chr 6
*STING1*	*ERIS*	KAL1288231	Chr 8
*TICAM1*	*TRIF; MyD88-3*	KAL1287077	Chr 8
*TIRAP*	*MYD88-2*	KAL1287636	Chr 5
*UNC93B1*	*UNC93; TLR signaling regulator*	KAL1286931	Chr 5
*TRAF1*	*TNF receptor associated factor 1*	KAL1288038	Chr 10
*TRAF2*	*TNF receptor associated factor 2*	KAL1288514	Chr 4
*TRAF3*	*TNF receptor associated factor 3*	KAL1286829	Chr 16
*TRAF4*	*TNF receptor associated factor 4*	KAL1287767	Chr 20
*TRAF5*	*TNF receptor associated factor 5*	KAL1287044	Chr 11
*TRAF6*	*TNF receptor associated factor 6*	KAL1287434	Chr 19
*TRAF7*	*TNF receptor associated factor 7*	KAL1286545	Chr 3
**Janus and other kinases**			
*JAK1*	*Janus kinase 1*	KAL1286939	Chr 9
*JAK2*	*Janus kinase 2*	KAL1287446	Chr 10
*JAK3*	*Janus kinase 3*	KAL1286737	Chr 8
*TYK2*	*Janus kinase JTK1*	KAL1286878	Chr 8
*IRAK1BP1*	*AIP70; SIMPL*	KAL1286883	Chr 14
*IRAK1*	*IRAK-1*	KAL1286446	Chr X
*IRAK2*	*IRAK-2*	KAL1287806	Chr 6
*IRAK3*	*IRAKM*	KAL1288117	Chr 3
*IRAK4*	*IRAK-4*	KAL1286989	Chr 3
*RIPK1*	*RIP-1*	KAL1287230	Chr 2
*RIPK2*	*RIP-2; CARD3*	KAL1288301	Chr 21
**STATs**	**Signal transducer/activation of transcription**		
*STAT1*	*CANDF7; IMD31A; IMD31B; IMD31C; ISGF-3; STAT91*	KAL1287623	Chr 2
*STAT2*	*IMD44; ISGF-3; P113; PTORCH3; STAT113*	KAL1288135	Chr 3
*STAT3*	*ADMIO; ADMIO1; APRF; HIES*	KAL1286482	Chr 20
*STAT4*	*DPMC; SLEB11*	KAL1287624	Chr 2
*STAT5A*	*MGF*	KAL1286483	Chr 20
*STAT5B*	*GHISID2*	KAL1286484	Chr 20
*STAT6*	*D12S1644; HIES6; IL-4-STAT; STAT6B; STAT6C*	KAL1288136	Chr 3
**Interferon pathway**			
*IFNA1*	*IFN-alpha 1*	KAL1286370	Chr 10
*IFNA2*	*IFN-alpha 2*	KAL1286361	Chr 10
*IFNA3*	*IFN-alpha 3*	KAL1286362	Chr 10
*IFNA4*	*IFN-alpha 4*	KAL1286377	Chr 10
*IFNB1*	*IFN-beta 1*	KAL1287444	Chr 10
*IFNE*	*IFN-epsilon*	KAL1287280	Chr 10
*IFNG*	*IFN-gamma*	KAL1288112	Chr 3
*IFNK*	*IFN-kappa*	KAL1287281	Chr 10
*IFNT1*	*IFN-tau 1*	KAL1286371	Chr 10
*IFNT2*	*IFN-tau 2*	KAL1286372	Chr 10
*IFNT3*	*IFN-tau 3*	KAL1286373	Chr 10
*IFNT4*	*IFN-tau 4*	KAL1286374	Chr 10
*IFNT5*	*IFN-tau 5*	KAL1286375	Chr 10
*IFNT6*	*IFN-tau 6*	KAL1286358	Chr 10
*IFNT7*	*IFN-tau 7*	KAL1286359	Chr 10
*IFNT8*	*IFN-tau 8*	KAL1286355	Chr 10
*IFNT9*	*IFN-tau 9*	KAL1286356	Chr 10
*IFNT10*	*IFN-tau 10*	KAL1286363	Chr 10
*IFNT11*	*IFN-tau 11*	KAL1286364	Chr 10
*IFNT12*	*IFN-tau 12*	KAL1286365	Chr 10
*IFNT13*	*IFN-tau 13*	KAL1286378	Chr 10
*LTA*	*Interferon B*	KAL1286623	Chr 2
*IFNAR1*	*Interferon-alpha R1*	KAL1286550	Chr 1
*IFNAR2*	*Interferon-alpha R2*	KAL1286551	Chr 1
*IFNGR1*	*CD119; IFN-gamma R1*	KAL1288382	Chr 14
*IFNGR2*	*IFN-gamma R2*	KAL1286552	Chr 1
*IFNLR1*	*IFN-lambda R1; IL-28 R1; IL-28 RA*	KAL1287147	Chr 2
*IL10RB*	*CD210B; CRFB4; IL-10R2; IL-10 RB; IL-28R*	KAL1286553	Chr 1
*IRF1*	*Interferon regulatory factor 1*	KAL1287074	Chr 8
*IRF2BP1*	*Interferon regulatory factor 2 BP1*	KAL1286769	Chr 15
*IRF2BP2*	*Interferon regulatory factor 2 BP2*	KAL1287784	Chr 6
*IRF2BPL*	*Interferon regulatory factor 2 BPL*	KAL1287051	Chr 13
*IRF2*	*Interferon regulatory factor 2*	KAL1287409	Chr 4
*IRF3*	*Interferon regulatory factor 3*	KAL1286770	Chr 15
*IRF4*	*Interferon regulatory factor 4*	KAL1286770	Chr 2
*IRF5*	*Interferon regulatory factor 5*	KAL1288356	Chr 12
*IRF6*	*Interferon regulatory factor 6*	KAL1287038	Chr 11
*IRF7*	*Interferon regulatory factor 7*	KAL1286920	Chr 5
*IRF8*	*Interferon regulatory factor 8*	KAL1287935	Chr 15
*IRF9*	*Interferon regulatory factor 9*	KAL1287250	Chr 13
*MAVS*	*IPS1; VISA; CARDIF*	KAL1287541	Chr 18
*IFRD1*	*Interferon dev regulator 1*	KAL1288353	Chr 12

Muskox orthologs of the pattern recognition receptors, interferons and intermediaries. See Supplementary Table S6 for the mitogen-activated protein kinases (MAPs), beta-defensins, interleukins, tumor necrosis factors (TNFs), and the chemokines.

### Muskox genes associated with Arctic adaptation

Supplementary Table S3 provides exon-level annotation, chromosome location, and citations of genes that are associated with Arctic adaptation^74^. These genes are involved in metabolic processes, including various aspects of brown adipose tissue (BAT)^92^ and circadian rhythm^93,94^. Supplementary Table S3 also includes candidate genes in the muskox previously identified^2^ as having convergent amino acid substitutions, are rapidly evolving, or that are under positive selection.

### Genes of the innate immunity pathways

There has been a marked increase in outbreaks of infectious disease among wildlife attributable to environmental disruptions from anthropogenic climate changes^79,80^. Arctic species are particularly vulnerable as pathogens invade new northern niches. The innate immune system is the first to respond to pathogens^95,96^. As a resource to support infection genomics and wildlife management, we provide gene annotations and accession numbers for the principal members of the innate immunity pathways in the muskox (Table 3 and Supplementary Tables S6 and S7).

Table 3 highlights the major classes of cell surface and intracellular innate receptors of the innate immunity system, and their principal signalling intermediaries in the muskox. These receptors include the Toll-like^97^, Nod-like^98^, C-type lectin^99^, and the RIG-I^100^ families of receptors that recognize pathogen-associated patterns or damage-associated molecular patterns. RIGI (DHX58) and IFIH1 (MDA5) are the principal sensors for nucleic acids from viral pathogens^101^. Once receptors are activated, signals are potentiated into the cell nucleus by specific adaptor molecules such as MYD88, TICAM1(TRIF), MAVS, and STING^102^, and a series of signalling intermediaries, notably the TRAFs, STATs and members of the IRAK, JAK^103^ and the mitogen-activated protein (MAPKs)^104^ families of kinases. Signals potentiated through MAPKs could also provide cross-talk with the adaptive immunity pathways, and the support of apoptosis or autophagy leading to the removal of damaged or infected cells. The 55 *MAPK* genes in muskox are provided in Supplementary Table S6.

Host responses upon receptor activation include the expression of interferons^105–107^ (Table 3), specific inflammatory cytokines^108^, chemokines^109,110^, and anti-microbial peptides such as the defensins^111^. Chemokines, notably CXCL7, CXCL9-11, CCL20, and CCL28 (Supplementary Table S6), appear to have direct antimicrobial activities similar to the defensins^109^. We identified 16 beta defensin genes in muskox (Supplementary Table S6). All paralogs of the large extended human interleukin-17 (*IL17A* to *IL17F*) and interleukin-17 receptor (*IL17RA* to *IL17RE*; *IL17REL*) families are present in muskox, pointing to a broad evolutionary conservation of all individual family members in the rapid response to infectious agents^112^. The chemokines, interleukins, and tumor necrosis factors and receptors involved in muskox innate immunity are presented in Supplementary Table S6.

### Genes of the major histocompatibility complex

The major histocompatibility complex (MHC) is the most important region in the genome with respect to adaptive and innate immunity^95,96,113–116^. In human, the MHC is located on the short arm of chromosome 6 (6p21.3). The MHC is particularly gene rich, occupying more than 7.5 Mbp of the genome, comprising several hundred genes encoding ligands, receptors, and other mediators and regulators of the inflammatory and immune responses. As such, the MHC plays a vital role in fighting infection by pathogenic agents. Apart from regulating immunity, the MHC may also have roles in reproduction and social behaviors, such as mate selection and kin recognition^117,118^.

The MHC is divided into three main regions loosely based on related structure or functions. Recently, the MHC has been extended to include the flanking genes^113^. The Class I region is located nearest to the telomere and includes the Class I leukocyte antigen presenting genes. These genes encode highly polymorphic cell surface receptor subunits, which when paired with a beta-2-microglobin subunit (B2M), the functional receptor present endogenous antigens processed from intercellular pathogens to T-lymphocytes (CD8 + T-cells). The MHC Class II region includes the Class II leukocyte antigen presentation genes, which also encode highly polymorphic cell surface receptor subunits. However, they differ from the Class I receptors in that they form functional receptors through heterodimers comprised of alpha and beta Class II receptor subunits. By contrast to the widespread expression of the Class I leukocyte receptors, Class II receptor expression is restricted to the professional antigen-presenting cells such as macrophages, dendritic and B cells, and present exogenous antigens to CD4 + helper T-cells. The Class III region contains genes encoding immune regulatory molecules, such as tumor necrosis factor (TNF), TNF family members, heat shock proteins, and complement factors.

**Figure 5:**
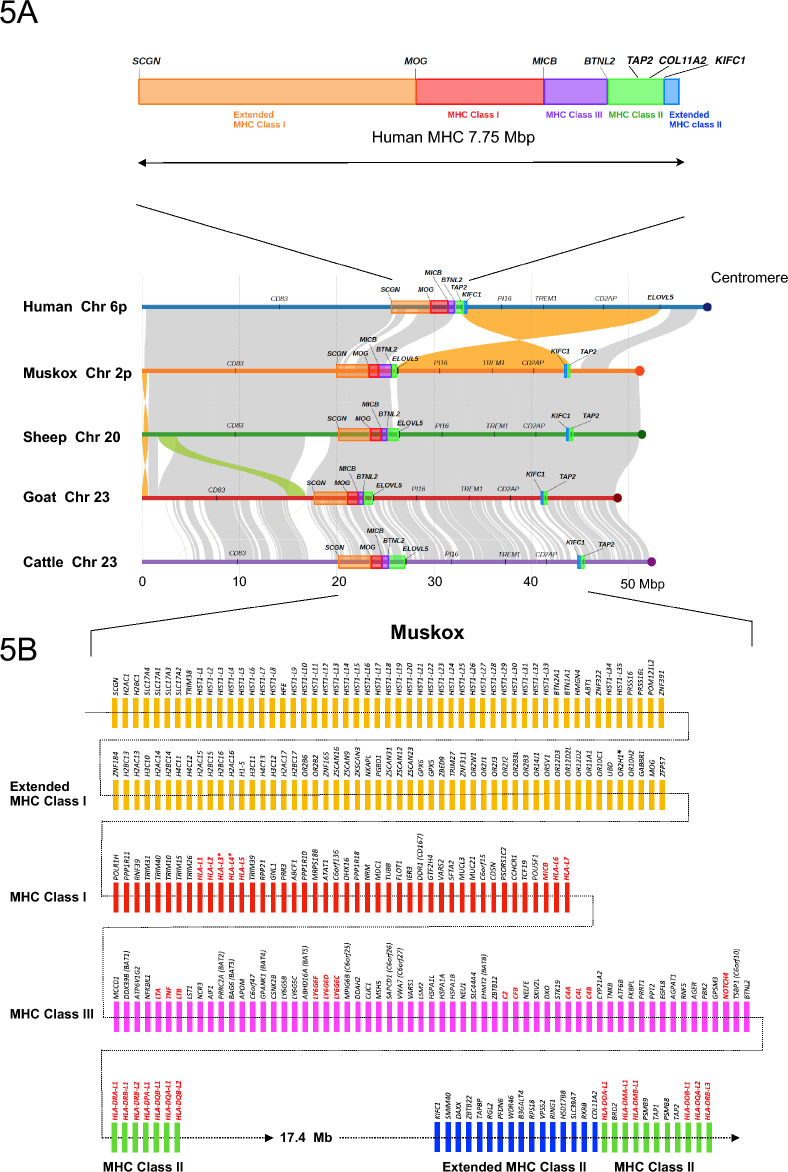
Major histocompatibility complex (MHC) on muskox chromosome 2. (**A**) Upper panel: Organization of the human MHC. Landmark genes serving as region boundaries are highlighted. Region lengths are drawn to scale. Lower panel: Comparison of the chromosome arm containing the MHC between human, muskox, sheep, goat and cattle was visualized using plotsr.^70^ Grey-shaded ribbons indicate regions of broad synteny, orange ribbons represent inversions, and green ribbons indicate translocations. When compared to human, the MHC Class II region in muskox, sheep, and cow is interrupted, where a segment is inverted in orientation from the rest of the cluster as well as being translocated 17.4 Mb toward the direction of the centromere. There is broad synteny across the chromosome arm among the Bovidae species, with cattle being the outgroup of the four. (**B**) Gene map of the 245 genes in the extended MHC in muskox. Class I and Class II leukocyte antigen presenting genes are in red type. Asterisk (*) denotes a pseudogene.

Figure 5A compares the chromosomal organizations of the MHC in human, muskox, sheep, goat and cow. The chromosome arms in the five species share extensive synteny along their lengths, with the cow less so as indicated by the shorter lengths of the synteny blocks. Overall, muskox MHC contains a similar number of principal genes and are organized similarly as in the other species. Figure 5B is a schematic of the functional MHC genes we annotated in the muskox. However, when compared to human MHC, a striking finding is that in muskox, sheep, and cow, the MHC Class II region is interrupted, where a large segment comprising the distal portion of the MHC Class II region through to the Extended MHC Class II region is inverted in orientation, resulting in the distal portion of the MHC Class II region being translocated 17.4 Mb away from the rest of the complex in the direction of the centromere. Otherwise, we observed a high degree of synteny in the overall organization of muskox chromosome 2 with human chromosome 6 in GRCh38.p14.

In the muskox MHC Class I region we identified five Class I leukocyte antigen presenting genes, *HLA-L1*, *HLA-L2*, *HLA-L5*, *HLA-L6* and *HLA-L7*. Two other genes, HLA-L3* and HLA-L4*, were predicted to be pseudogenes based on the presence of in-frame termination codons, which were verified using Illumina short reads. *MICA* and *MICB* in human are adjacent stress-inducible genes related to the MHC Class I leukocyte antigen presenting genes^119^, but MICA and MICB use KLRK1 (NKG2D) as the co-receptor subunit instead of B2M^120^. *KLRK1* (*CD314, NKG2-D*) and *B2M* co-receptor genes are located on muskox chromosomes 3 and 13, respectively. Interesting, we observed a tandem duplication of *B2M* on muskox chromosome 13. The duplicated gene appears to be functional and was assigned the symbol *B2ML.* In the muskox, we only find *MICB.* Since the two genes might have overlapping functions, the absence of *MICA* in the muskox might be mitigated by redundancy; however, its absence could impair the muskox’s response to certain stressors.

In the muskox MHC Class II region, we identified thirteen Class II leukocyte antigen presenting genes: *HLA-DRA-L1*, *HLA-DRB-L1*, *HLA-DRB-L2*, *HLA-DPA-L1*, *HLA-DQB-L1*, *HLA-DQA-L1*, *HLA-DQB-L2*, *HLA-DOA-L1*, *HLA-DMA-L1*, *HLA-DMB-L1*, *HLA-DOB-L1*, *HLA-DQA-L2*, and *HLA-DRB-L3*. As denoted by the A and B designations in their assigned gene symbols, six genes encode potential alpha-receptor subunits and seven genes encode potential beta-receptor subunits.

The MHC Class III genes are generally heterogeneous in structure. None present antigens to T-cells, but most are involved in some other aspects of immune regulation or inflammation^121,122^. We identified and annotated 61 genes in the muskox MHC Class III region, including members of the TNF family^123,124^, the Lymphocyte Antigen-6 family members^125^, and the members of the complement cascade.^126^ Compared to human, we observed a tandem duplication of the *C4* gene in the muskox. The second gene was assigned the symbol *C4L*. Including the 121 genes in the extended Class I and Class II regions, the final number of annotated genes in the muskox extended MHC is 245. Exon-level annotations for these genes are provided in Supplementary Table S4.

Two CD antigens are encoded by the MHC, *CD167* (*DDR1*) and *CD33*7 (*NCR3*). CD (Cluster of Differentiation) molecules or antigens are cell surface markers, many of which are receptors or ligands involved in activation or inhibitory pathways.^127^ As such, many CD antigens are biomarkers of, or are participants in, infectious disease. A list of the several hundred annotated CD antigens in the muskox can be found in Supplementary Table S7.

### Genetic diversity of muskox MHC

We have shown the muskox MHC region is similar in structure and overall genomic organization to the other Bovidae, indicating no potential deficiencies in muskox due to gross deletions or arrangements in the MHC region. The number of MHC Class I and Class II genes in the muskox is also similar to many other mammals, suggest that at face value, the muskox is immune-competent in interactions with T-cells. Genetic diversity within a population is viewed to be important in evolutionary ecology and conservation biology^128–131^. We thus examined the genetic (polymorphic) diversity of the MHC genes in muskox.

In an early study of the muskox, no genetic diversity was found using an exon 2 probe that spanned the MHC *DRB* locus (corresponding to *HLA-DRB-L1* in the present study)^132^. This early result points to a lack of MHC diversity in muskox. However, the study was constrained by the small size of the study cohort, and by the limited sensitivity of amplicon sequencing that targeted only a single exon. With the complete characterization of muskox MHC combined with the use of whole genome re-sequencing and mapping-based variant calling methods^133–135^ we could now overcome the sensitivity and throughput issues associated with previous amplicon sequencing attempts. To test the feasibility of this approach, we took advantage of whole-genome short-reads from a recent population survey of the muskox^31^ to provide an initial assessment of the genetic diversity of all the genes in the MHC of the muskox. These reads are available through NCBI SRA and are comprised of sets of Illumina reads with an average 14× genome coverage of individuals from 18 distinct populations of muskoxen across their native range. For our study, we downloaded reads from one representative specimen from each population. We included reads from a 21,100-year-old individual from Siberia from which inferences could be made of the muskox’s demographic history.

**Figure 6:**
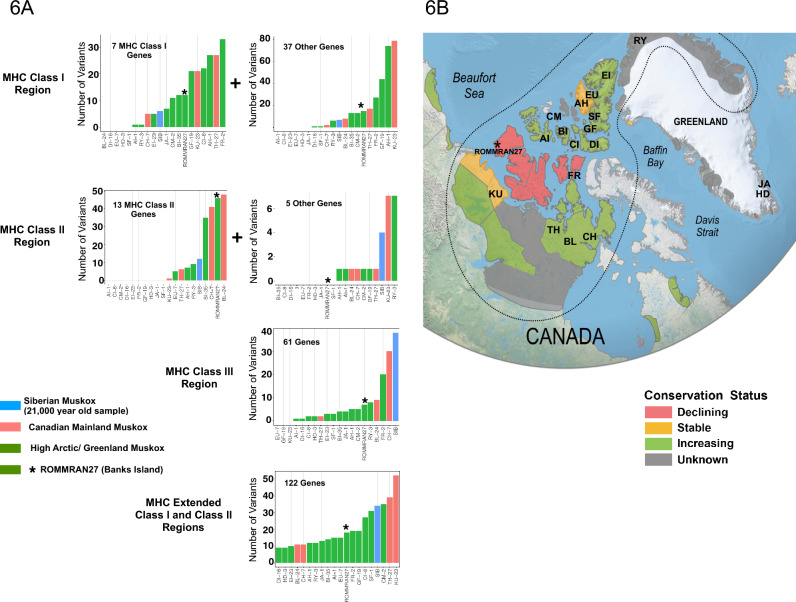
Gene diversity in the MHC region of muskoxen across various geographic locations. (**A**) Illumina reads of 20 muskoxen, including a 21,000-year-old individual from Siberia, were used to tabulate variants in the coding region of the 245 genes in the MHC (see Material and Methods). ROMMRAN27, from which we have assembled the genome is denoted by an asterisk (*). The two-letter prefix is a geographic abbreviation. The prehistoric sample from Siberia is designated SIB. (**B**) Geographic origins of the 19 muskox samples in northern Canada and Greenland. The Siberian sample is not depicted.

Reads from individual muskox samples were mapped onto our newly constructed reference assembly, O.moschatus_RAN27-v1.0, and variant calls were made from the mapped reads against this reference genome. Figure 6A presents the variant calls made for each muskox specimen across the coding regions of genes in the MHC regions (see Fig. 5B for a schematic of the gene organization in the muskox). All variant calls were found to be single base substitutions. We did not observe any insertions or deletions among the calls. The specimen from Banks Island, ROMMRAN27, from which DNA was used to assemble the reference genome is denoted by an asterisk (*). Variant calls made for ROMMRAN27 were heterozygous Single Nucleotide Polymorphisms (SNPs) present in our sample. The two-letter prefix for each sample is a geographic identifier denoting the origin of the 18 muskox specimen shown in Fig. 6B. The prehistoric sample from Siberia is designated SIB.

Variant calls spanning the coding regions of the 245 genes in the MHC across all 20 muskox samples are tabulated in Supplementary Table S5. It is likely the calls presented in Supplementary Table S5 are an under-representation, since we adopt stringent mapping criteria in an effort to minimize false positive variant calls. The sensitivity of a short-read mapping approach to call variants is highest for the regions of the genome that are free of major mapping impediments, such as the presence of highly repetitive sequences and other features associated with genomic regions of low complexity. Historically, the MHC region was renowned for the presence of such impediments, which could not be fully resolved until the recent advent of using long reads with better mapping specificity for variant calling^136,137^. However, until the implementation of using long reads to make variant calls for the muskox, the present use of shortreads might result in significant false-negative calls, but it would still provide a useful first look at the genetic diversity in this region of the genome.

From the initial set of variants depicted in Fig. 6A, there appears to be no clear and consistent regional pattern, despite that some populations have been through human-caused bottlenecks, while others have been through climate-caused ones. Quite possibly, low population sampling has hampered a more complete assessment of the species. For example, there are no representations from Victoria Island, and Banks Island is represented only by our reference specimen, which seemed to be neither particularly invariant nor have a high number of variants compared to those from other Arctic islands. Interestingly, the prehistoric sample from Siberia, SIB, does not consistently have the highest number of variants across all six groups of genes.

The MHC Class I and Class II genes function to present processed antigens to CD8+ T-cells and CD4+ helper T-cells, respectively, and are critical for mounting an immune response. Table 4 summarizes variant calls for the seven MHC Class I genes, and the thirteen MHC Class II genes across the twenty muskox samples. Column 3 depicts the number of sets of unique combinations of single base variants (variant profile) for each gene across the 19 modern muskox specimens. However, in the absence of phasing information, such as long reads, RNA-seq reads, or other genetic information, to establish the two haplotypes, we cannot reconstruct the two alleles from each variant profile with certainty. Nevertheless, these profiles provide a direct indication, albeit a lower limit, of the coding diversity of the muskox MHC.

**Table 4:** Number of variants in the MHC Class I and Class II genes across 20 muskox samples of different geographic locations.

Category	Gene	Unique combinations of variants in 19 modern specimens	Siberia	Canada mainland				Canada Arctic Islands												Greenland		
			SIB	BL-24	CH-7	KU-23	TH-27	*ROM	AI-1	AH-1	BI-35	CI-8	CM-2	DI-16	EI-23	EU-7	FR-2	GF-19	SF-1	HD-3	JA-1	RY-3
MHC Class I	*HLA-L1*	9			5	21	8	3		17	3	12	3				12	11				
MHC Class I	*HLA-L2*	4	3				13			1		1					9					
MHC Class I	HLA-L3*	8					5	6			7		6				12	1			7	
MHC Class I	HLA-L4*	5	2				1	2			2		2		5							
MHC Class I	*HLA-L5*	1																				
MHC Class I	*HLA-L6*	1	1																			
MHC Class I	*HLA-L7*	2						1	1													1
MHC Class II	*HLA-DRA-L1*	3		2	2			1		2												2
MHC Class II	*HLA-DRB-L1*	7		7	6		5	7		5						5						6
MHC Class II	*HLA-DRB-L2*	2	3		1			1														
MHC Class II	*HLA-DPA-L1*	2	1	2	2			2			2											
MHC Class II	*HLA-DQB-L1*	3	3	26	2			26			26											
MHC Class II	*HLA-DQA-L1*	1	3																			
MHC Class II	*HLA-DQB-L2*	3	1	9	9			9			7											
MHC Class II	*HLA-DOA-L1*	2																				1
MHC Class II	*HLA-DMA-L1*	2		1	1		1															
MHC Class II	*HLA-DMB-L1*	1																				
MHC Class II	*HLA-DOB-L1*	1																				
MHC Class II	*HLA-DQA-L2*	2		1		1																
MHC Class II	*HLA-DRB-L3*	1	1																			

Variant calls for the seven MHC Class I genes and thirteen MHC Class II genes were performed in as described in the Materials and Methods. The assembled sample ROMMRAN27 (*ROM) is in bold. Pseudogenes are designated by an asterisk after the gene name. Column 3 depicts the number of sets of unique combinations of single base variants (variant profile) for each gene across the 19 modern muskox specimens.

Even though the small sample size precludes an in-depth analysis for the muskox, some general observations can be made. For instance, *HLA-L1* and *HLA-L2* appear to be particularly polymorphic across many samples. Mainland muskoxen, with the exception of the Helena Island sample (sample BI), and our Banks Island sample (ROMMRAN27), are generally more diverse than samples from the other Arctic islands, especially those from Eastern Greenland (samples JA and HD). Interestingly, Class I pseudogenes, HLAL3* and HLA-L4*, accumulated many variants principally across the Arctic Island samples, none of which however restore the coding sequence to create a functional gene. Finally, we observed that *HLA-L5, HLA-L6, HLA-DQA-L1, HLA-DQ-L1, HLA-DMB-L1, HLA-DQB-L1,* and *HLA-DRB-L3* are markedly free of variants across all samples, when compared to our reference specimen, ROMMRAN27.

Genetic diversity in the MHC is believed to impact wildlife health and conservation^128–131^. In contrast to early work pointing to low or no genetic diversity in the muskox MHC^132^, our tabulation of variants across multiple specimens showed a significant diversity across the MHC. Although an extensive direct gene-to-gene comparison could not be made, at least qualitatively in selective regions that could be compared, the diversity of the muskox MHC appears to be similar to the level reported for the reindeer, another Arctic ruminant under threat^138^. While the muskox has been shown to have the lowest genome-wide heterozygosity reported in an ungulate^31^ (Fig. 3), this lack of genetic diversity is not necessarily extended to the MHC region. This finding, along with the muskox having no overt signs of inbreeding depression in native populations^31^, is consistent with the view of Teixeira and Huber^139^ that argues against the perceived importance of neutral genetic diversity over functional genetic diversity for the conservation of wild populations and species.

### Conservation management

The present study provides genomics tools that will advance muskox conservation and management in at least three ways. First, the creation of a high-quality reference genome provides a resource to develop genetic markers that could be used estimate gene flow, effective population size, the timing of extirpation, and to monitor progress of future recolonization efforts. As shown by our initial survey of diversity of the MHC in the muskox, whole genome reference-assisted re-sequencing is a sensitive and powerful tool to identify markers. In the immediate future, the use of re-sequencing is likely to be used in a discovery phase, where selective cohorts are re-sequenced to generate a comprehensive set of informative sequence markers, after which these markers would be interrogated on lower cost and higher throughput genotyping panels that are constructed for specific applications.

Second, the genomic tools especially the annotated genes will contribute to management and conservation. Muskox population structure and connectivity can now be assessed from both a neutral and adaptive basis, as currently muskox populations are delimited based on hunting areas. The annotated genes can contribute to measure connectivity between populations and for understanding the dynamics of expanding muskox distribution on the mainland or options for translocations to rescue depleted populations. Regional populations can be sampled to assess for local adaptive loci identification from the annotated genes, and regional planning undertaken to conserve local adaptations. However, there are still considerable uncertainties in the present muskox population numbers and trends, thereby limiting the usefulness of the new genomics tools. In their recent review^19^, Cuyler and colleagues have acknowledged these shortcomings and have proposed increased frequency of muskox surveys, standardizing monitoring protocols, and the inclusion other informative metrics.

Finally, the remarkable super-fine under-wool of the muskox has garnered much attention for its economic potential, and has driven some early attempts to domesticate the muskox to improve yield and ease of qiviut harvest^12–15^. While the role of domestication as a driver for conservation and management of endangered species is controversial^140–142^, it could be one facet of an integrated adaptive solution that is locally implemented. However, domestication is a long and difficult process, but the genetics of domestication are relatively well understood for livestock^143–148^. Although muskox domestication has been attempted, it has not yet succeeded^149–151^. Efforts were too small-scale and brief to allow the selection for particular traits, although the muskox became tame. As a first step to support a possible future marker assisted selection scheme^152–154^ for the muskox, we provide exon-level annotation of genes that have contributed to livestock domestication (Supplementary Table S8) and desirable commercial traits such as meat and dairy production, wool quality, prolificity, and heat tolerance^155–159^ (Supplementary Table S9).

**Figure 7:**
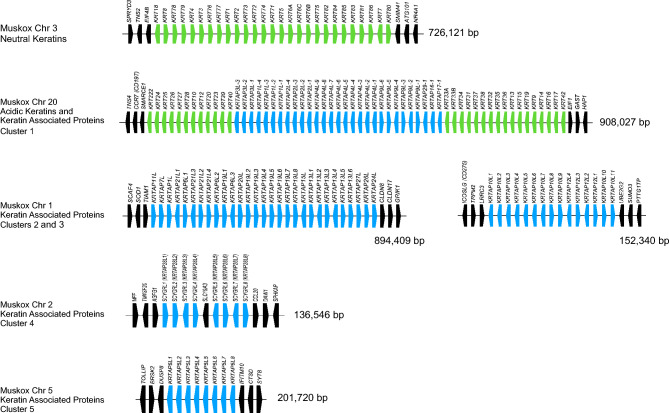
Schematic of Muskox keratin and keratin-associated protein gene clusters. Gene clusters are depicted in the same orientation as in the assembly. Genes flanking the clusters are depicted by black boxes. Keratin genes are depicted by green boxes. Keratin-associated protein genes are depicted by blue boxes. The direction of transcription is indicated by the box outdent. Gene annotations can be found in Supplementary Table S10.

Given the high market price of qiviut, the genetic signatures for qiviut may become useful in the future, especially if the genetics of its production differ regionally with different muskox populations. To this end, Fig. 7 and Supplementary Table S10 show the organization and annotation of the principal keratin and keratin-associated protein gene clusters in muskox. Keratins and the keratin-associated proteins are conserved across multiple species with family members encoding the principal structural components of hair and wool^160–163^. Importantly, variants of these genes have been shown to associate with wool quality, and are potential selection markers for livestock improvements^164–167^.

Arctic conservation management can be complex with intersecting and at times conflicting environmental, cultural and socioeconomic issues^90,168,169^. However, co-management (i.e., joint management between Indigenous people and government) and adaptive management^169,170^, where multiple integrated solutions are differentially implemented locally, can help navigate many of those complexities. In particular, a co-management regime gives a clear voice to local communities in the balancing of cultural traditions, and costs incurred by environmental protection can be offset against the potential returns from developing economic values of muskoxen, including meat and leather production, sport hunting, tourism, and production of qiviut.

In conclusion, we report a chromosomal-level assembly of a muskox from Banks Island in the Canadian Arctic Archipelago with a view toward genomics-based approaches for the management and conservation of the muskox, with relevant benefits to Inuit communities and muskox conservation.

## Supplementary Information

Supplementary Information.

Supplementary Tables.

## Data Availability

Sequences described for the muskox have been submitted to GenBank and other public archives and repositories. This study has been assigned BioProject number PRJNA1071053 to facilitate data dissemination. Accessions for muskox genes depicted in the manuscript are listed in the relevant Supplementary Tables.
