# Supplementary material for: Chromosomal-level reference genome assembly of muskox (Ovibos moschatus) from Banks Island in the Canadian Arctic, a resource for conservation genomics
Source: Sci Rep. 2024 Sep 16;14:21023. doi: 10.1038/s41598-024-67270-9 (PMC11405533; doi:10.1038/s41598-024-67270-9)
Supplement: Supplementary file 1 — Supplementary Information. [file 41598_2024_67270_MOESM1_ESM.docx]

**Chromosomal-Level Reference Genome Assembly of a Banks Island Muskox (Ovibos moschatus), a Resource for Conservation Genomics**

Si Lok, Timothy NH Lau, Brett Trost, Amy HY Tong, Tara Paton, Richard F Wintle, Mark D Engstrom, Anne Gunn, Stephen W Scherer

**Supplementary Figures**

Supp. Figure S1 DNA profile and length distribution of sequence reads

Supp. Figure S2 Contamination assessment for the muskox assembly

**Supplementary Tables**

­

Supp. Table S1 Report of repetitive content in the muskox assembly by RepeatMasker

Supp. Table S2 Genes scored as BUSCO Fragmented or Missing

Supp. Table S3 Genes associated with arctic adaptation

Supp. Table S4 Gene list of the MHC region

Supp. Table S5 Variant table of MHC region across 20 muskox samples

Supp. Table S6 Genes of the innate immunity pathways

Supp. Table S7 CD antigens gene list

Supp. Table S8 Genes associated with domestication

Supp. Table S9 Genes associated with commercially desirable traits

Supp. Table S10 Keratin and keratin-associated proteins

Supp. Table S11 Accession number of Pečnerová, P. *et al*. data set

**Supplementary Figure S1.** **DNA profile and Length distribution of sequence reads.** a) Length distribution profile on the Agilent Tape Station of the genomic DNA extracted from the muskox kidney sample. Based on the internal 100 bp calibration marker, the sampled DNA used in the study has a peak length of 22,553 bp. b) Length distribution of PacBio CLRs generated on the Sequel II. Reads below 2 kb were discarded. c) Read count, N50 and estimated genome coverage of the reads used in the assembly were tabulated, along with a breakdown for each length category. A total of 16,756,911 reads were used, summing up to 207,603,336,852 bases, representing an estimated genomic coverage of 71.6x.

**Supplementary Figure S2.** **Contamination Assessment for the Muskox Assembly.** The assembled and polished muskox assembly was assessed for foreign DNA contaminants by blasting it against NCBI’s non-redundant Nucleotide (nt) database in decreasing phylogenetic distance from muskox. The filters in sequential order are (1) selected genome assemblies of the sheep, takin and goat, (2) members of the Caprinae family, (3) other members of the Pecora family, and (4) organisms outside the Pecora family. (5) non-Pecora hits are further categorized into different lineages (a) to (l), with their definitions based on NCBI’s Taxonomy categories: (a) Bacteria = Bacteria (NCBI:txid2); (b) Protist = Eukaryota (txid759) minus [Viridiplantae (txid33090), Rhodophyta (taxid2763), Metazoa (txid33208), and Fungi (txid4751)]; (c) Plants = Viridiplantae (txid33090); (d) Fungi = Fungi (txid751); (e) Invertebrates = Metazoa (txid33208); (f) Fishes = Actinopterygii (txid7898); (g) Amphibians = Amphibia (txid8292); (h) Mammals = Mammalia (txid40674) ; (i) Reptiles = Sauropsida (txid8457) minus Aves (txid8782); (j) Birds = Aves (txid8782); (k) Viruses = Viruses (txid10239); (l) Others = None of the above. “No significant blast hits” filters out blast hits with length <100 bp. Blast hits that are >50% repeat-masked are not counted when hit outside the Reference genomes. A contig 25,142 bp in length was found to be a contaminant from the b-proteobacterial family of *Comamonadaceae*, and has been removed from the final assembly.

**References for Supplementary Tables**

**Supplementary Table S3**

Bal, N., Maurya, S., Sopariwala, D. *et al.* Sarcolipin is a newly identified regulator of muscle-based thermogenesis in mammals. *Nat. Med.* **18**, 1575–1579; 10.1038/nm.2897 (2012).

Cardona, A. *et al.* Genome-Wide Analysis of Cold Adaptation in Indigenous Siberian Populations. *PLOS ONE* **9**, e98076; 10.1371/journal.pone.0098076 (2014).

Cooper, M. P., Uldry, M., Kajimura, S., Arany, Z. & Spiegelman, B. M. Modulation of PGC-1 Coactivator Pathways in Brown Fat Differentiation through LRP130. *Journal of Biological Chemistry* **283**, 31960–31967 (2008).

Fumagalli, M. et al., Greenlandic Inuit show genetic signatures of diet and climate adaptation. *Science* **349**,1343-1347(2015).

Hallmark, B. *et al.* Genomic Evidence of Local Adaptation to Climate and Diet in Indigenous Siberians. *Molecular Biology and Evolution* **36**, 315–327 (2019).

Igoshin, A. V., Gunbin, K. V., Yudin, N. S. & Voevoda, M. I. Searching for Signatures of Cold Climate Adaptation in TRPM8 Gene in Populations of East Asian Ancestry. *Front. Genet.* **10**, (2019).

Ikeda, K., Shiba, S., Horie-Inoue, K. *et al.* A stabilizing factor for mitochondrial respiratory supercomplex assembly regulates energy metabolism in muscle. *Nat Commun* **4**, 2147; 10.1038/ncomms3147 (2013).

Li, M. *et al.* Convergent molecular evolution of thermogenesis and circadian rhythm in Arctic ruminants. *Proceedings of the Royal Society B: Biological Sciences* **290**, 20230538; 10.1098/rspb.2023.0538 (2023).

Liu, Y. *et al*. Genes expression related to Intramuscular Fat deposition in muscles of small Tail Han sheep. *Journal of Animal and Veterinary Advances* **11,** 3969-3977 (2012).

Lynch, V. J. *et al*. Elephantid genomes reveal the molecular bases of woolly mammoth adaptations to the Arctic. *Cell Reports* **12,** 217-228 (2015).

Moltke, Ida, *et al*. A common Greenlandic TBC1D4 variant confers muscle insulin resistance and type 2 diabetes. *Nature* **512,** 190-193 (2014).

Nam, M. & Cooper, M. P. Role of energy metabolism in the brown fat gene program. *Front. Endocrinol.* **6,** 104 (2015).

Nikanorova, A. A. *et al*. The Role of Nonshivering Thermogenesis Genes on Leptin Levels Regulation in Residents of the Coldest Region of Siberia. *International Journal of Molecular Sciences* **22,** 4657; 10.3390/ijms22094657 (2021).

Nowack, J., Giroud, S., Arnold, W. & Ruf, T. Muscle Non-shivering Thermogenesis and Its Role in the Evolution of Endothermy. *Front. Physiol.* **8,** 899; 10.3389/fphys.2017.00889 (2017).

Reynolds, A. W. *et al*. Comparing signals of natural selection between three Indigenous North American populations. *Proceedings of the National Academy of Sciences* **116,** 9312–9317 (2019).

Samaniego Castruita, J. A., Westbury, M. V. & Lorenzen, E. D. Analyses of key genes involved in Arctic adaptation in polar bears suggest selection on both standing variation and de novo mutations played an important role. *BMC Genomics* **21,** 543; 10.1186/s12864-020-06940-0 (2020).

**Supplementary Table S6**

Abraha, R. Review on the role and biology of cytokines in adaptive and innate immune system. *Archives of Veterinary and Animal Sciences* **2** (2020).

Aristizábal, B. & González, A. Chapter 2 Innate immune system. *In* Autoimmunity from bench to bedside. Anaya, J.-M. *et al*. (eds). El Rosario University Press, Bogatoa, Colombia (2013).

Bousoik, E. & Alibadi, H. M. Do we know jack about JAK? A closer look at JAK/STAT signaling. *Front. Oncol.* **8,** 287; 10.3389/fonc.2018.00287 (2018).

Chen, H. & Jiang, Z. The essential adapters of innate immune signaling. *Protein Cell* **4,** 27-39 (2013).

Esche, C. *et al*. Chemokines: Key players in innate and adaptive immunity. *J. Invest. Dermatol.* **125,** 615-628 (2005).

McFadden, G. *et al*. Cytokine determinants of viral tropism. *Nature Reviews Immunology* **9,** 645-655; 10.1038/nri2623 (2009)

McNab, F. *et al.* Type I interferons in infectious disease. *Nat. Rev. Immunol.* **15**, 87-103; 10.1038/nri3787 (2015).

Sedy, J. *et al.* Tumor necrosis factor superfamily in innate immunity and inflammation. *Cold Spring Harb. Perspect. Biol.* **7,** a016279; 10.1101/cshperspect.a016279 (2015).

Sokol, C. L. & Luster, A. D. The chemokine systems in innate immunity. *Cold Spring Harb. Perspect. Biol.* **7,** a016303; 10.1101/cshperspect.a016303 (2015).

Xu, D. & Lu, W. Defensins: A Double-Edged Sword in Host Immunity. *Frontiers in Immunology* **11,** 538653; 10.3389/fimmu.2020.00764 (2020).

**Supplementary Table S8**

Ahmad, H. I. *et al*. The domestication makeup: evolution, survival, and challenges. *Front. Ecol. Evol.* **8,** 103; 10.3389/fevo.2020.00103 (2020).

Alberto, F. J. *et al.* Convergent genomic signatures of domestication in sheep and goats. *Nat. Comm.* **9,** 813; 10.1038/s41467-018-03206-y (2018).

Dou, M. *et al*. A missense mutation in RRM1 contribute to animal tameness. *Sci. Adv.* **9,** eadf4068; 10.1126/sciadv.adf4068 (2023).

Zheng, Z. *et al*. The origin of domestication genes in goats. *Sci. Adv.* **6,** eaaz5216; 10.1126/sciadv.aaz5216 (2020).

**Supplementary Table S9**

Abou-Naga, A. M. *et al.* Genome-wide analysis identified candidate variants and genes associated with heat-stress adaptation in Egyptian sheep breeds. *Front. Genet.* **13,** 898522; 10.3389/fgene.2022.898522 (2022).

Ahbara, A. *et al.* Regions and genes associated with fat deposition and tail morphology in Ethiopian indigenous sheep. *Front. Genet.* **9,** 699; 10.3389/fgene.2018.00699 (2019).

Archana, P. R. *et al.* Role of heat shock proteins in livestock adaptation to heat stress. *J. Dairy Vet. Anim. Res.* **5(1)**, 13-19 (2017).

Arora, R., Yadav, H. S. & Yadav, D. K. Identification of novel single nucleotide polymorphisms in candidate genes for mutton quality in Indian sheep. *Animal Molecular Breeding* **4**, 1-5; 10.5376/amb.2014.04.0001 (2014).

Baazaoui, I., Bedhiaf-Romdhani, S., Mastrangelo, S. & Ciani, E. Genome-wide analyses reveal population structure and identify candidate genes associated with tail fatness in local sheep from a semi-arid area. *Animal* **15,** 100193; 10.1016/j.animal.2021.100193 (2021).

Bakhtiarizadeh, M. R. & Alamouti, A. A. RNA-Seq based genetic variant discovery provides new insights into controlling fat deposition in the tail of sheep. *Sci Rep* **10,** 13525; 10.1038/s41598-020-70527-8 (2020).

Becker, G. M., Woods, J. L., Schauer, C. S., Stewart, W. C. & Murdoch, B. M. Genetic association of wool quality characteristics in United States Rambouillet sheep. *Front. Genet.* **13,** 1081175; 10.3389/fgene.2022.1081175 (2023).

Cao, Y. *et al*. Genome-wide association study of body weights in Hu sheep and population verification of related single-nucleotide polymorphisms. *Front. Genet.* **11,** 588; 10.3389/fgene.2020.00588 (2020).

Chiang, C. *et al*. Essential Role for Sonic hedgehog during Hair Follicle Morphogenesis. *Developmental Biology* **205,** 1–9 (1999).

Collier, R. J. *et al*. Invited review: genes involved in the bovine heat stress response. *J. Dairy Sci.* **91**, 445-454 (2008).

Demars, J. *et al.* Genome-Wide Identification of the Mutation Underlying Fleece Variation and Discriminating Ancestral Hairy Species from Modern Woolly Sheep. *Molecular Biology and Evolution* **34,** 1722–1729 (2017).

Dicks, P., Russel, A. J. F. & Lincoln, G. A. The role of prolactin in the reactivation of hair follicles in relation to moulting in cashmere goats. *Journal of Endocrinology* **143,** 441-448 (1994).

Dikmem, S. *et al*. Genome-wide association mapping for identification of quantitative trait loci for rectal temperature during heat stress in Holstein cattle. *PLoS one* **8(7)**, e69202 (2013).

Ebrahimi, F., Gholizadeh, M., Rahimi-Mianji, G. & Farhadi, A. Detection of QTL for greasy fleece weight in sheep using a 50 K single nucleotide polymorphism chip. *Trop Anim Health Prod* **49,** 1657–1662 (2017).

Farhadi, S. *et al*. Comprehensive Gene Expression Profiling Analysis of Adipose Tissue in Male Individuals from Fat- and Thin-Tailed Sheep Breeds. *Animals* **13,** 3475; 10.3390/ani13223475 (2023).

Farhadi, S., Shodja Ghias, J., Hasanpur, K., Mohammadi, S. A. & Ebrahimie, E. Molecular mechanisms of fat deposition: IL-6 is a hub gene in fat lipolysis, comparing thin-tailed with fat-tailed sheep breeds. *Archives Animal Breeding* **64,** 53–68 (2021).

Gavran, M., Antunović, Z., & Gantner, V. Candidate genes associated with economically important traits of sheep-a review. *Agriculturae Conspectus Scientificus* **86,** 195-201 (2021).

Gebreselassie, G., Berihulay, H., Jiang, L. & Ma, Y. Review on Genomic Regions and Candidate Genes Associated with Economically Important Production and Reproduction Traits in Sheep (*Ovies aries*). Animals **10,** 33; 10.3390/ani10010033 (2020).

Georges, M. *et al*. Harnessing genomics information for livestock improvement. *Nat. Rev. Genet.* **20,** 135-156 (2019).

Ghildiyal, K. *et al*. Selection signatures for fiber production in commercial species: A review. *Animal Genetics* **54,** 3–23 (2023).

Gutiérrez-Gil, B. *et al.* High-resolution analysis of selection sweeps identified between fine-wool Merino and coarse-wool Churra sheep breeds. *Genetics Selection Evolution* **49,** 81; 10.1186/s12711-017-0354-x (2017).

Habimana, V. *et al.* Genes and models for estimating genetic parameters for heat tolerance in dairy cattle. *Front. Genet.* **14,** 1127175; 10.3389/fgene.2023.1127175 (2023).

Hariyono, D. N. H. & Prihandini, P. W. Association of selected gene polymorphisms with thermotolerance traits in cattle – A review. *Animal Bioscience* **35,** 1635–1648 (2022).

Hector, T. E. *et al*. Infection burdens and virulence under heat stress: ecological and evolutionary considerations. *Phil. Trans. R. Soc.* B **378**, 20220018 (2023).

Hosseini, S. F., Bakhtiarizadeh, M. R. & Salehi, A. Meta-analysis of RNA-Seq datasets highlights novel genes/pathways involved in fat deposition in fat-tail of sheep. *Front. Vet. Sci.* **10,** 1159921; 10.3389/fvets.2023.1159921 (2023).

Hu, T. *et al.* miR-143 Targeting CUX1 to Regulate Proliferation of Dermal Papilla Cells in Hu Sheep. *Genes* **12,** 2017; 10.3390/genes12122017 (2021).

Hussain, T. *et al.* Cattle be on two mind states: an overview of heat stress tolerance in cattle. *Intl. J. Agric. Biol.* **29,** 133-140 (2023).

Jiang, D. I. *et al*. Genome-wide association studies on the wool staple crimp frequency in Chinese Merino Sheep (Xinjiang Type). *Xinjiang Agricultural Sciences* **52,** 2129 (2015).

Knapik, J., Ropka-Molik, K. & Pieszka, M. Genetic and Nutritional Factors Determining the Production and Quality of Sheep Meat – A Review. *Annals of Animal Science* **17,** 23–40 (2017).

Lan, X. Y. *et al.* Twelve novel SNPs of the goat POU1F1 gene and their associations with cashmere traits. *Small Ruminant Research* **85,** 116–121 (2009).

Li, M. *et al.* Convergent molecular evolution of thermogenesis and circadian rhythm in arctic ruminants. *Proceedings of the Royal Society B* **290,** 20230538; 10.1098/rspb.2023.0538 (2023).

Li, M.-H., Tiirikka, T. & Kantanen, J. A genome-wide scan study identifies a single nucleotide substitution in ASIP associated with white versus non-white coat-colour variation in sheep (*Ovis aries*). *Heredity* **112,** 122–131 (2014).

Li, S. *et al.* Variation in the ovine KAP6-3 gene (KRTAP6-3) is associated with variation in mean fibre diameter-associated wool traits. *Genes* **8,** 204; 10.3390/genes8080204 (2017).

Li, W. *et al.* CRISPR/Cas9 mediated loss of FGF5 function increases wool staple length in sheep. *The FEBS Journal* **284,** 2764–2773 (2017).

Liang, B. *et al*. Two mutations at KRT74 and EDAR synergistically drive the fine-wool production in Chinese sheep. *Journal of Advanced Research* **57,** 1–13 (2024).

Ling, Y. H. *et al.* Identification of complete linkage disequilibrium in the DSG4 gene and its association with wool length and crimp in Chinese indigenous sheep. *Genet. Mol. Res* **13** , 5617-5624 (2014).

Liu Y. *et al.* Genes expression related to Intramuscular Fat deposition in muscles of small Tail Han sheep. *Journal of Animal and Veterinary Advances* **11,** 3969-3977 (2012).

Lomba, Y. L. Review on quantitative trait locus (QTLs) for yield and quality of milk, meat, fibre, eggs and their application. *International Journal of Animal Science, Husbandry and Livestock Production* **6,** 285-291 (2020).

Luo, R. *et al.* GLIS1, a potential candidate gene affect fat deposition in sheep tail. *Mol. Biol. Rep.* **48,** 4925–4931 (2021).

Ma, S. *et al.* Transcriptome analysis reveals genes associated with wool fineness in merinos. *PeerJ* **11,** e15327; 10.7717/peerj.15327 (2023).

Mastrangelo, S. *et al.* Novel and known signals of selection for fat deposition in domestic sheep breeds from Africa and Eurasia. *PLOS ONE* **14,** e0209632; 10.1371/journal.pone.0209632 (2019).

McManus, C. M. *et al*. Heat stress effects on sheep: Are hair sheep more heat resistant? *Theriogenology* **155**, 157-167 (2020).

Megdiche, S., Mastrangelo, S., Ben Hamouda, M., Lenstra, J. A. & Ciani, E. A. Combined Multi-Cohort Approach Reveals Novel and Known Genome-Wide Selection Signatures for Wool Traits in Merino and Merino-Derived Sheep Breeds. *Front. Genet.* **10,** 468521; 10.3389/fgene.2019.01025 (2019).

Mendoza, M. N., Raudsepp, T., Alshanbari, F., Gutiérrez, G. & Ponce de León, F. A. Chromosomal Localization of Candidate Genes for Fiber Growth and Color in Alpaca (Vicugna pacos). *Front. Genet.* **10,** 433376; 10.3389/fgene.2019.00583 (2019).

Mohamadipoor Saadatabadi, L. *et al.* Signature selection analysis reveals candidate genes associated with production traits in Iranian sheep breeds. *BMC Vet. Res.* **17,** 369; 10.1186/s12917-021-03077-4 (2021).

More, M. *et al.* Genome-Wide Association Study of Fiber Diameter in Alpacas. *Animals* **13,** 3316; 10.3390/ani13213316 (2023).

Mu, F. *et al.* Structural Characterization and Association of Ovine Dickkopf-1 Gene with Wool Production and Quality Traits in Chinese Merino. *Genes* **8,** 400; 10.3390/genes8120400 (2017).

Negahdary, M., Majdi, S., & Hajihosseinlo, A. Genetic effect of IGF1, PIT1 and Leptin genes on wool weights in Makooei sheep. *Electron J. Biol.* **10,** 46-51 (2014).

Otto, P. I. *et al*. Genome-wide association studies for heat-stress response in *Bos Taurus* x *Bos indicus* crossbred cattle. *J. Dairy Sci.* **102**, 8148-8158 (2019).

Sadeghi, M., Jalil-Sarghale, A. & Moradi-Shahrbabak, M. Associations of POU1F1 gene polymorphisms and protein structure changes with growth traits and blood metabolites in two Iranian sheep breeds. *J Genet* **93**, 831–835 (2014).

Sejian, V. *et al*. Review: adaption of animal to heat stress. *Anim.* **12**, s431-s444 (2018).

Shi, R. et al. Identification of key genes and signaling pathways related to Hetian sheep wool density by RNA-seq technology. *PLOS ONE* **17,** e0265989; 10.1371/journal.pone.0265989 (2022).

Stewart, C., Hamilton, G., Marshall, C. J. & Stevenson, T. J. Transcriptome analyses of nine endocrine tissues identifies organism-wide transcript distribution and structure in the Siberian hamster. *Sci. Rep.* **12,** 13552; 10.1038/s41598-022-16731-0 (2022).

Sun, W., Ma, S. & Ma,Y. On hair follicle development and wool production traits in sheep: a review. *International Journal of Agriculture and Biology* **25,** 450-454 (2021)

Tian, W. *et al.* Effects of ginsenoside Rg1 on glucose metabolism and liver injury in streptozotocin-induced type 2 diabetic rats. *Genet. Mol. Res.* **16,** gmr16019463 (2017).

Vijayakumar, P. *et al.* Whole-genome comparative analysis reveals genetic mechanisms of disease resistance and heat tolerance of tropical *Bos indicus* cattle breed. *Genome* **65**, 241-254 (2022).

Wang, F. H. *et al.* Genome wide association study of fleece traits in Inner Mongolia Cashmere goats. *Animal Genetics* **52,** 375–379 (2021).

Wang, X. *et al.* Identification of key genes in sheep fat tail evolution Based on RNA-seq. *Gene* **781,** 145492; 10.1016/j.gene.2021.145492 (2021).

Wang, Z. *et al.* Genome-Wide Association Study for Wool Production Traits in a Chinese Merino Sheep Population. *PLOS ONE* **9,** e107101; 10.1371/journal.pone.0107101 (2014).

Weller, J. I., Golik, M., Seroussi, E., Ezra, E. & Ron, M. Population-Wide Analysis of a QTL Affecting Milk-Fat Production in the Israeli Holstein Population. *Journal of Dairy Science* **86,** 2219–2227 (2003).

Worku, D. *et al.* Candidate genes associated with heat stress and breeding strategies to relieve its effects in dairy cattle: a deeper insight into the genetic architecture and immune response to heat stress. *Front. Vet. Sci.* **10,** 1151241; 10.3389/fvets.2023.1151241 (2023).

Xu, S. S. *et al.* Genome-wide association analysis identifies the genetic basis of fat deposition in the tails of sheep (*Ovis aries*). *Animal Genetics* **48,** 560–569 (2017).

Yue, Y. *et al.* Exploring Differentially Expressed Genes and Natural Antisense Transcripts in Sheep (Ovis aries) Skin with Different Wool Fiber Diameters by Digital Gene Expression Profiling. *PLOS ONE* **10,** e0129249; 10.1371/journal.pone.0129249 (2015).

Yurchenko, A. A. *et al*. High-density genotyping reveals signatures of selection related to acclimation and economically important traits in 15 local sheep breeds from Russia. *BMC Genomics* **20,** 294; 10.1186/s12864-019-5537-0 (2019).

Zeng, L. *et al.* Genes related to heat tolerance in cattle-A review. *Anim. Biotech.* **34,** 1840-1848 (2023).

Zhang, C. *et al.* Identification of the key proteins associated with different hair types in sheep and goats. *Front. Genet.* **13,** 993192; 10.3389/fgene.2022.993192 (2022).

Zhang, T. *et al.* Genome-wide association studies revealed candidate genes for tail fat deposition and body size in the Hulun Buir sheep. *Journal of Animal Breeding and Genetics* **136,** 362–370 (2019).

Zhang, X. *et al.* Alteration of sheep coat color pattern by disruption of ASIP gene via CRISPR Cas9. *Sci. Rep.* **7,** 8149; 10.1038/s41598-017-08636-0 (2017).

Zhao, B. *et al.* Comprehensive transcriptome and methylome analysis delineates the biological basis of hair follicle development and wool-related traits in Merino sheep. *BMC Biology* **19,** 197; 10.1186/s12915-021-01127-9 (2021).

Zhao, B. *et al.* Integration of a single-step genome-wide association study with a multi-tissue transcriptome analysis provides novel insights into the genetic basis of wool and weight traits in sheep. *Genetics Selection Evolution* **53,** 56; 10.1186/s12711-021-00649-8 (2021).

Zhao, H. *et al.* Genome-wide association studies detects candidate genes for wool traits by re-sequencing in Chinese fine-wool sheep. *BMC Genomics* **22,** 127; s12864-021-07399-3 (2021).

Zhu, C., Li, N., Cheng, H. & Ma, Y. Genome wide association study for the identification of genes associated with tail fat deposition in Chinese sheep breeds. *Biology Open* **10,** bio054932; 10.1242/bio.054932 (2021).

**Supplementary Table S11**

Pečnerová, P. et al. Population genomics of the muskox’s resilience in the near absence of genetic variation. *Mol. Ecol.* **33,** e17205; 10.1111/mec.17205 (2024).
